# Bio-Compatible Ca-BDC/Polymer Monolithic Composites Templated from Bio-Active Ca-BDC Co-Stabilized CO_2_-in-Water High Internal Phase Emulsions

**DOI:** 10.3390/polym12040931

**Published:** 2020-04-17

**Authors:** Xule Yang, Youwei Hao, Liqin Cao

**Affiliations:** Key Laboratory of Oil and Gas Fine Chemicals, Ministry of Education & Xinjiang Uygur Autonomous Region, Xinjiang University, Urumqi 830046, China; xly0202@yeah.net (X.Y.); aywhao@163.com (Y.H.)

**Keywords:** metal organic framework, Ca-BDC, CO_2_-in-water emulsions, high internal phase emulsions, Pickering emulsifiers, interconnected macro-porous MOF/polymer composites, 3D hierarchical porous MOFs integral monoliths

## Abstract

Because of the nontoxic solvents contained in CO_2_-in-water emulsions, porous polymer composites templated from these emulsions are conducive for bio-applications. Herein, bio-active rod-like calcium-organic framworks (Ca-BDC MOFs, BDC= 1,4-benzenedicarboxylate anion) particles co-stabilized CO_2_-in-water high internal phase emulsion (C/W HIPE) in the presence of polyvinyl alcohol (PVA) is first presented. After curing of the continuous phase, followed by releasing CO_2_, integral 3D macro-porous Ca-BDC monolith and Ca-BDC/Poly(2-hydroxyethyl methacrylate-*co*-acrylamide) HIPEs monolithic composites [Ca-BDC/P(AM-*co*-HEMA)HIPEs] with open-cell macro-porous structures were successfully prepared. The pore structure of these porous composite can be tuned by means of tailoring the Ca-BDC dosage, carbon dioxide pressure, and continuous phase volume fractions in corresponding C/W HIPEs. Results of bio-compatibility tests show that these Ca-BDC/P(AM-*co*-HEMA)HIPEs monoliths have non-cytotoxicity on HepG2 cells; also, the *E. coli* can grow either on the surfaces or inside these monoliths. Furthermore, immobilization of *β*-amylase on these porous composite presents that *β*-amylase can be well-anchored into the porous polymer composites, its catalytic activity can be maintained even after 10 cycles. This work combined bio-active MOFs Ca-BDC, bio-compatible open-cell macroporous polymer PAM-*co*-HEMA and green C/W HIPEs to present a novel and facile way to prepare interconnected macro-porous MOFs/polymer composites. Compared with the existing other well-known materials such as hydrogels, these porous composites possess well-defined tunable pore structures and superior bio-activity, thereby have promising applications in bio-tissue engineering, food, and pharmaceutical.

## 1. Introduction

Macroporous structure in bio-materials for tissue engineering scaffolds and enzyme carriers etc. are of great significance [1,2]. The existing well-known materials such as hydrogel [3], which are used largely in tissue engineering for synthetic bio-materials fabrication or in drug delivery systems for pharmaceuticals are restricted by the unadjustability and irregularity of their pore structure. Currently, researchers have tried to prepare interconnected macro-porous polymer materials by a variety of methods [4,5,6]. Among them, emulsion templating has become increasingly prominent.

High internal phase emulsion (HIPE) is such an emulsion, in which internal phase fractions account for 0.74 or more. Especially, oil-in-water (O/W) HIPEs, in which the oil phases are dispersed phases, take aqueous solutions as continuous phases, after curing of continuous phases, hydrophilic, or amphiphilic polyHIPEs with porous structure and high porosity can be prepared by removing dispersed phases [7]. By simply adjusting the formulas and polymerization conditions of these HIPEs, performances transformations of corresponding polyHIPEs can be easily achieved to cater to applications in wider fields [1,3,7,8,9]. Nevertheless, conventional O/W HIPEs requires a large amount of surfactants and organic solvents, post-treatments are cumbersome, and residual surfactants and organic solvents are mostly not only toxic but also environmentally unfriendly, which restrict their applications in certain practical fields, such as food [10] and pharmaceutical [11].

Attractively, carbon dioxide (CO_2_) is non-toxic, non-polluting, non-flammable, and inexpensive, easily available naturally. It can act as green solvents replacing conventional organic solvents. Supercritical or compressed CO_2_ (scCO_2_) have moderate critical temperature and pressure (31.1 °C and 7.38 MPa), and their solvation ability can be easily tuned by changing their temperature and pressure [12,13]. Moreover, its entraining effects can introduce biologically active ingredients and functionally small molecules into functional materials and can extract small organic molecules from the composite components [14,15]. Especially, CO_2_ instead of organic solvents used in O/W HIPEs produce scCO_2_-in-water high internal phase emulsions (C/W HIPEs), which have been applied in preparing a series of hydrophilic porous polyHIPEs under the actions of effective surfactants [16,17,18] through a greenway. However, because of the weak solvation ability of CO_2_ as well as high interfacial tension and the large free energy of CO_2_-water interfaces, most surfactants, which are available for O/W emulsions, maybe not well capable for C/W emulsions [17]. Currently, emulsifiers for C/W emulsion systems have been discovered and reported including hydrocarbon surfactants [18] and solid particles [19,20,21,22]. Fluorine-containing surfactants are currently the best CO_2_-philic type surfactants, but they are expensive, toxic, and non-degradable, which make them impossible to obtain industrial applications [18].

Owing to the simple compositions (metal centers and organic linkers) and designable ordered topologies, MOFs generally feature large specific areas, super functionalities, high thermal stability, and permanent porosity, which endow MOFs excellent performances in high-end applications [23,24,25,26,27]. Whereas the MOFs crystals obtained by the conventional methods are generally powders, easy to agglomerate due to excessive free energy caused by rest empty coordination, which is not conducive for them to disperse and reuse, resulting in wasting and polluting. Fortunately, considering the adjustable compositions and unique structures, MOFs can be designed with specific functions and amphiphilic properties to act as Pickering emulsifiers to emulsify different emulsions, including CO_2_-in-water emulsions [28,29,30]. Moreover, MOFs/polymer composites can be templated from MOFs emulsified CO_2_-in-water emulsions [31,32,33] and symbolized as MOFs/PolyHIPEs. These MOFs/PolyHIPEs composites combined the properties of metal coordination polymers with the nature of a macroporous macromolecular network to improve material performances [32,34]. Compared with traditional high internal phase emulsions for synthesizing polymer porous composites, the superiorities of MOFs emulsified C/W HIPE templates are that: (1) The MOFs particles play roles in not only well emulsifying C/W HIPEs but also providing MOFs/PolyHIPEs with enhanced properties such as improved mechanical performances [35], catalytic [36], electrical [37], as well as magnetic properties and thermal conductivity [38]; (2) MOFs/PolyHIPEs can be easily obtained by releasing CO_2_, no solvent residues; (3) pore structures of MOFs/PolyHIPEs are adjustable because of the designable MOFs and tunability of CO_2_ solvent. Moreover, because of the green solvents involved in the MOFs emulsified C/W HIPEs, these emulsions are more suitable for the synthesis of bio-compatible or bio-active porous composites. But generally the MOFs used such as manganese, nickel, copper, and cobalt-based MOFs [30], are toxic, which restricts their applications in bio-fields. Therefore it is necessary to find bio-compatible or bio-active MOFs to prepare bio-compatible or bio-active MOFs/polymer porous composites to make the most of MOFs and these green emulsions.

Calcium-terephthalate (Ca-BDC MOFs), consisting of bio-friendly metal centers Ca^2+^ ions and organic linkers benzene-1,4-dicarboxylic acid (BDC), is a kind of alkaline earth metal organic framework based on rod-like building blocks, advantageous for biological applications [39,40] because of their non-toxicity and relatively low densities. The Ca-BDC’s negative zeta potential of −12.5 mV in the water at room temperature and contact angle of 25.5° through aqueous phase indicates relative hydrophilicity. Compared with the hydrogels generally used in tissue engineering, the mineralization of porous soft 3D material can provide them with plenty of sites for the growth of bone cells and play a significant role in nutrient transport when the material templates from the Ca-BDC co-stabilized HIPEs. However, there are few works to integrate bio-active MOFs into porous polymer composites via CO_2_-in-water emulsions.

Herein, we first propose the utilization of the rod-like bio-active Ca-BDC and PVA to co-stabilized C/W HIPEs and employed these green emulsions as a template to synthesize Ca-BDC/PolyHIPEs porous composites with open-cell macro-porous structure. Additionally, the effects of formulas of these C/W HIPEs on the pore structure, mechanical performances, and other properties were also investigated. Also, the effects of Ca-BDC/P(AM-*co*-HEMA)HIPEs on cell proliferation of HepG2 cells and *E. coli*s growth, as well as *β*-amylases immobilization in these Ca-BDC/P(AM-*co*-HEMA)HIPEs were performed to confirm the potential bio-application of these materials.

## 2. Materials and Methods

### 2.1. Materials

Calcium nitrate tetrahydrate (Ca(NO_3_)_2_·4H_2_O, 99%) was purchased from Tianjin Shengmiao Chemistry Company (Tianjin, China). Benzene-1,4-dicarboxylic acid (H_2_BDC, ≥98%) was supplied by J&K Scientific Ltd. (Beijing, China). *N,N′*-Dimethylformamide (DMF) and polyvinyl alcohol (PVA, *M*_w_< 27000 g/mol) were obtained from Sigma-Aldrich (Beijing, China). Anhydrous methanol (≥99.5%), anhydrous ethanol (≥99.5%), 2-Hydroxyethyl methylacrylate (HEMA, ≥98%), acrylamide (AM, ≥98%), *N,N′*-methylene bis(acrylamide) (MBA, ≥96%), potassium persulfate (KPS, 99.5%), cyclohexane (99.5%) were supplied by Tianjin Chemical Reagent Co. Ltd. (Tianjin, China). CO_2_ with a purity of ≥99.9% was supplied by Urumqi Gas Factory (Urumqi, China). Deionized water was freshly prepared and used for all the experiments. Polymerization reactions were carried out in a 100 mL high-pressure stainless steel reactor (Datong Production Autoclave Container Manufacturing Co., Ltd., Dalian, China). The pressure in the reactor was measured in the range of 0–30 MPa using a pressure gauge (Raral Instrument Co., Ltd., Chengdu, China). A collecting magnetic heating stirrer (Jiangsu Jinyi Instrument Technology Co., Ltd., Jintan, China) was used to blend the reaction system. All chemicals of reagent grade or better above were used as received.

### 2.2. Synthesis of Ca-BDC MOF

A reaction mixture of benzene-1,4-dicarboxylic acid (H_2_BDC, 0.664 g, 4 mmol), calcium nitrate tetrahydrate (Ca(NO_3_)_2_·4H_2_O, 1.89 g, 8 mmol), deionized water (20 mL), and *N,N′*-dimethylformamide (DMF, 60 mL) was stirred for 30 min at room temperature to form a homogeneous solution with a pH value of 6.40. The solvothermal reactions were carried out by heating the reaction mixtures in a 100-mL Teflon-lined digestion bombs to 150 °C under autogenous pressure for 24 h, followed by slowly cooling at 6 °C·h^−1^ to room temperature. Colorless crystals of Ca-BDC were washed with methanol solution (95 vol %), filtered off by centrifugation at a speed of 5000 r·min^−1^, dried at 50 °C in an oven [41].

### 2.3. Ca-BDC MOF Emulsification Test

To test the emulsifying ability of Ca-BDC particles, the role of different amount of Ca-BDC MOF in O/W HIPEs formation was initially determined according to literatures [30]. The as-synthesized MOF (1, 3, 5, and 7 wt %, with respect to water) was respectively added into four 10 mL stoppered plastic bottle containing 5 mL mixture of 4 mL cyclohexane and 1 mL H_2_O to form the Ca-BDC solely stabilized high internal phase emulsions (referred to as Ca-BDC solely stabilized O/W HIPEs). In addition, a little amount of PVA (1 wt %, with respect to water) was respectively added to four O/W HIPEs containing certain amount of Ca-BDC particles (0, 1, 3, 5 wt %, with respect to water) to form another four high internal phase emulsions respectively (referred to as Ca-BDC co-stabilized O/W HIPEs). These O/W HIPEs were formed by shaking with hands, and their stability was determined by direct observation. These O/W HIPEs microstructure were observed by the confocal laser scanning microscopy (CLSM, LSM 800, ZEISS, Jena, Germany) using a 543 nm laser to excite the samples, and the aqueous phase was stained with Rhodamine B prior to the test. Moreover, two 20 mL uniform aqueous solutions, one containing just a certain amount of (5 wt %, with respect to water) Ca-BDC particles, another one with a certain amount of PVA (1 wt %, with respect to water) and a certain amount of (5 wt %, with respect to water) Ca-BDC particles, were formed in a high-pressure stainless reactor (Datong Production Autoclave Container Manufacturing Co., Ltd., Dalian, China) by magnetic stirring to be the continuous phase, and desired amount of CO_2_ (50 g) was charged to be the disperse phase. After keeping stirring for 2 h to form CO_2_-in-water HIPE (C/W HIPE), the reactors were transferred into the refrigerator to freeze dry at −20 °C for a whole night, and then release CO_2_ in the refrigerator. After freezing drying the frozen monolith, pure Ca-BDC (templated from Ca-BDC solely stabilized C/W HIPE) and integral PVA modified 3D Ca-BDC macroporous monoliths conformed closely to interior of reactor were produced. Note that all the volume fractions of the internal phase were 80% in this work.

### 2.4. Preparations of Ca-BDC/PolyHIPEs Monolithic Composites

An aqueous solution containing desired amount of emulsifier Ca-BDC particles, polyvinyl alcohol (PVA), monomers acrylamide (AM) and 2-hydroxyethyl methylacrylate (HEMA), cross-linker *N,N′*-methylene bis(acrylamide) (MBA), and initiator potassium persulfate(KPS) was transferred into 100-mL high pressure stainless reactor, followed by stirring for 30 min to form a homogenous solution. Then the reactor was sealed and immersed in an ice water bath. After draining the air in the kettle by a flow of CO_2_ for a few minutes, the reactor was charged with the desired amount of CO_2_ (40–70 g). A homogenous milk-white C/W emulsion was formed by magnetic stirring for 2 h at a fixed speed (1000 rpm·min^−1^) at room temperature. Subsequently, the reactor was immediately transferred to a 60 °C thermostat water bath, curing for 8 h, stirring simultaneously. Ultimately, after cooling the reactor at room temperature, CO_2_ in the kettle was released, and white monoliths were obtained and named as Ca-BDC/P(AM-*co*-HEMA)HIPEs. As a comparison, resulting polyHIPEs contain no monomer HEMA and Ca-BDC particles were symbolized asCa-BDC/P(AM)HIPE and P(AM-*co*-HEMA)HIPE, respectively. According to processes described above, a dozen porous polymer monoliths were prepared and abbreviated as PxCyWz, x, y, and z represent the content of Ca-BDC, CO_2_, and water in corresponding C/W HIPEs, respectively (see details in Table S1).

### 2.5. Characterization

Fourier transform infrared spectrometer (FTIR, Vertex 70, Bruker Optics, Ettlingen, Germany) was used to determine the chemical constituents of as-prepared specimens. The morphologies of the various specimens were observed using LEO 1430VP and Hitachi S-4800 field-emission scanning electron microscope (SEM, Hitachi High-Tech Corporation, Tokyo, Japan), samples were mounted on aluminum studs using adhesive graphite tape and sputter coated with approximately 10 nm of gold before analysis.

Powder X-ray diffraction (PXRD, Bruker D8 X, Bruker Ltd., Karlsruhe, Germany) was used to identify the phase compositions of the prepared specimens. The working conditions of PXRD were Cu-Kα radiation via a rotating anode at 40 kV and 50 mA. The data were collected in steps of 5° min^−1^ and the range of scattering angles (2θ) from 5° to 80°.

The confocal laser scanning microscopy (CLSM) images of the HIPEs were obtained on a LSM 800 microscope (ZEISS, ZEISS, Jena, Germany) using a 543 nm laser to excite the samples, and the aqueous phase was stained with rhodamine B prior to the test.

Contact angle measurements were performed at 25 °C using a contact angle meter (HARKE-SPCAX1, Beijing Harco Test Instrument Factory, Beijing, China) by placing a water droplet on the surface of the MOF tablets. The PVA-modified Ca-BDC particles, which were collected after stirring the aqueous solution containing as-prepared Ca-BDC particles and PVA for 30 min, were also used to test the contact angle. Each system was measured three times, and the contact angle was the average of the three results.

### 2.6. Mechanical Properties Analysis

Static mechanical tester (H5KT, Tinius Olsen, Horsham, PA, US) was used to measure the mechanical properties of the freshly obtained PolyHIPEs (3 g specimens contains about 2 g H_2_O) and swollen PolyHIPEs. The corresponding compression strain–stress curves were plotted to determine the mechanical characteristic of as-prepared PolyHIPEs. These specimens were prepared with 1 cm high cylinders with 2 cm in diameter. All experiments were repeated five times at 5 mm/s, and the results were averaged.

### 2.7. Determination of Pore and Pore Throats Size Distributions

The SEM images of all porous matrixes were used to determine the porous structure of as-prepared polyHIPEs [42,43]. In the SEM images, the cavity remained after removal of CO_2_ droplets were defined as pores, and the “windows” to interconnect pores were defined as pore throats. In practice, several micrographs taken at a magnification that allowed us to view about 50 pores (or pore throats) at a time were collected from different areas chosen at random within the exposed surface of the specimen. Images of each sample were imported into the program Image J (National Institutes of Health, Bethesda, MD, US), and the diameters of all these pores and pore throats (from a few to several hundred) were measured and used to determine the number distributions of pores and pore throats as well as their average values (D and d). The levels of the interconnectivity of pores were defined as the ratios of average pore throats size and average pores size (d/D), respectively.

### 2.8. Bio-Compatibility Test

HepG2 cells standard growth curve determination: HepG2 cells with a confluence of 90% were prepared, and a 5 × 10^4^ cells/mL single cell suspension was prepared in complete medium and inoculated into 96-well plates every 24 h (100 μL/well, 5 replicate wells, i.e., 5 × 10^3^ cells/hole). After 8 days, the cell culture medium was carefully cleaned, and 100 μL 10% CCK-8 solutions were added to each well, followed by placing these 96-well plates in an incubator at 37 °C, and the OD values of resulting CCK-8 solution at 450 nm wavelength were measured after 1 h. Standard growth curves were plotted, and the logarithmic growth phases of the cells were determined for subsequent experiments.

Effects of as-synthesized scaffolds on HepG2 cell proliferation: The porous composites were cut into granules, which were immersed in 70% ethanol solution for 24 h, washed with sterile PBS solution for three times, and irradiated with ultraviolet rays for 2 h for sterilization. HepG2 cells with a confluence of 90% were harvested, and the cells were digested with trypsin to prepare 5 × 10^4^ cells/mL single cell suspension in complete medium, and resulting single cell suspension were inoculated into 96-well plates (100 μL/well, 3 replicate wells, i.e., 3 × 10^3^ cells/hole). After the cells were attached to the well wall, two as-processed materials were added into 96-wells based on the different dosages (low concentration: 5 mg/mL, medium concentration: 10 mg/mL, high concentration: 20 mg/mL). Three replicates of each group were placed in an incubator at 37 °C and 5% CO_2_. After incubation for 48 h and 96 h, 1 μMcalcein was added, allowed to stand for 30 min, and viable cells were observed under a fluorescence microscope to evaluate the growth states of HepG2 cells in the incubation solution. Then the medium was discarded, 100 μL of the configured 10% CCK-8 solution was added to each well, and incubation was continued in the incubator. After 1 h, the OD values of the resulting CCK-8 solutions at 450 nm were measured with a microplate reader to detect cell proliferation.

Effect of as-synthesized scaffolds on *E. coli* growth: 4 to 5 mL of the *E. coli* solutions cultured at 37 °C for 18 to 20 h were taken and inoculated into a 500-mL conical flask containing 100 to 120 mL of nutrient broth medium. It was shaken, and divided evenly into three dry and sterile 100-mL Erlenmeyer flasks, then placed in a constant temperature oven at 37 °C. Then P_0.75_C_50_W_15_ and Ca-BDC were added to two of the three Erlenmeyer flasks. The remaining Erlenmeyer flask was used as a blank. Then 1 mL of the solution was sampled at intervals and the optical density OD values were measured at 550 nm and the growth curves were drawn.

### 2.9. Enzyme Immobilization

Immobilization of *β*-amylases: 0.25 g of prepared porous composite was added to the *β*-amylase solution diluted with phosphate buffer, and after magnetic stirring in a room temperature water bath for a certain time, the mixtures were filtered. The precipitates were washed several times with phosphate buffer, filtered, and dried to obtain immobilized *β*-amylase. Residual liquid and washing liquid were used to measure the OD values at 595 nm, according to Coomassie Blue Colorimetry. The residual protein concentration was determined through a standard curve of the protein absorbance-concentration standard curve:

(1) $$y=0.427x+0.0049\;\left(R^{2}=0.9985\right)$$

Protein adsorption capacity was defined as the protein content adsorbed by 1 g as-prepared PolyHIPEs:

(2) Qe=(C0−Ce)Vm

where *Q*_e_ is the equilibrium adsorption capacity (mg/g); *C*_0_ is the concentration of enzyme solution at time t = 0 (mg/mL), *C*_e_ is the concentration of enzyme solution at time of adsorption equilibrium t = e (mg/mL), *V*(mL) is the volume of resulting reaction solution, and m(g) is the amount of specimens used to immobilize enzyme.

*β*-Amylase activity assay: UV-visible spectroscopy combined with starch-iodine colorimetry was used to determine the catalytic activity of *β*-amylases using a starch solution as a substrate. In detail, 5 mL of phosphate buffer solution was added to 20 mL of 2 mg/mL starch solution (prepared on the same day), and preheated for 5 min at the test temperature. Then, 1 mL of *β*-amylase solution or 50 mg of immobilized *β*-amylases sample was added to the starch solution and reacted for 5 min; 1 mL of the reaction mixture was added into 5 mL of dilute iodine solution. After shaking, the concentrations of residual starch were determined by checking the standard curve of starch based on the OD values at a wavelength of 560 nm:

y = 1.2878x + 0.0456 (*R*^2^ = 0.9990) (3)

Enzyme catalytic activity was calculated by measuring the amount of residual starch after the enzyme reaction, and the amount of oxidized starch of 1 min of the enzyme was 1 vitality unit:

(4) $$U=(C_{0}-C_{t})V/t$$

where U is the enzyme vitality (mg/s); *C*_0_ is the concentration of starch solution at time t = 0 (mg/mL), *C*_t_ is the concentration of starch solution at a reaction time t = t (mg/mL), *V* (mL) is the volume of resulting reaction solution.

First, the immobilized *β*-amylases, prepared under different conditions, were used to test their catalytic activity in a starch solution with pH = 5 at room temperature, to determine the optimal immobilization condition. Then the catalytic activity of immobilized *β*-amylases and free *β*-amylases were measured in series of starch solutions with pH range of 4 to 8, and in the temperature range of 40 to 80 °C to determine their environment resistance. Taking the maximum catalytic activity as 100%, the relative enzyme activities were calculated based on the maximum catalytic activity. Immobilized *β*-amylases were stored at room temperature, and enzyme catalytic activity under the optimum catalytic conditions was determined at intervals. Immobilized *β*-amylases were also recovered and reused by washing, suction filtration, and drying. The relative catalytic activity after storage and reuse were calculated according to the activity of freshly immobilized *β*-amylase as 100%. In all processes, the averages of OD values were taken after measuring three times in parallel.

## 3. Results and Discussion

### 3.1. HIPEs Emulsified by Ca-BDC

**Figure 1:**
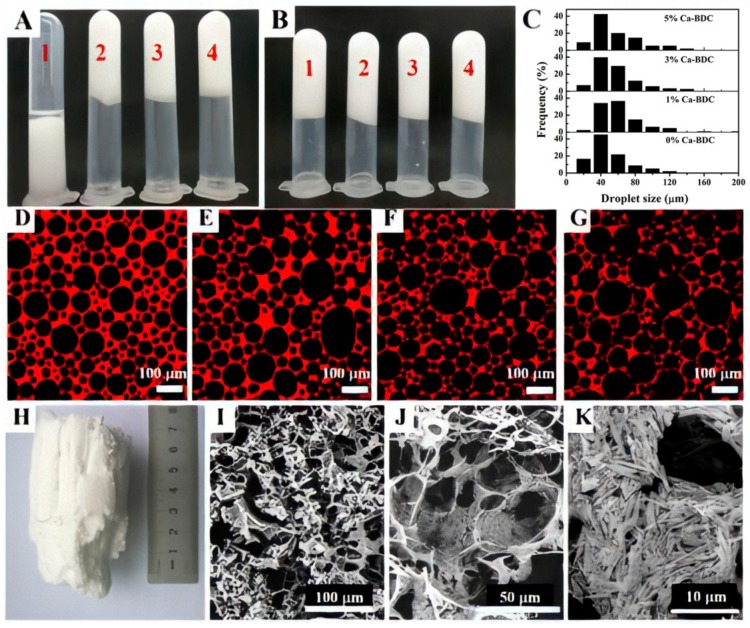
(**A**) Calcium-terephthalate (Ca-BDC) solely emulsified oil-in-water (O/W) high internal phase emulsions (HIPEs) (1), (2), (3), and (4) containing 1, 3, 5, and 7 wt % of Ca-BDC in absence of polyvinyl alcohol (PVA), respectively; (**B**) Ca-BDC particles co-stabilized O/W HIPEs (1), (2), (3), and (4) containing 0, 1, 3, and 5 wt % of Ca-BDC in presence of PVA, respectively; (**C**) is the droplets size distribution chart; (**D**), (**E**) and (**F**), (**G**) are the confocal laser scanning microscopy (CLSM) images of Ca-BDC co-stabilized O/W HIPE (1), (2), (3), and (4); (**H**) is the photograph of integral 3D PVA-modified Ca-BDC monolith templated from Ca-BDC co-stabilized CO_2_-in-water (C/W) HIPE in the presence of PVA; (**I**) and (**J**) are the SEM images of the microstructure of integral 3D PVA-modified Ca-BDC monolith; (**K**) is a magnified view of (**J**) to show the separated ultra-fine Ca-BDC nano-rods.

Calcium, an essential element of life, is the main inorganic component of human bones and teeth, accounts for about 1.4% of the body’s mass, and is closely related to the physiological activities associated with enzymes or proteins. Therefore bio-active Ca-BDC particles were synthesized according to the literature reported [41] (see more details in **ESI**) and were intended to be incorporated into bio-compatible porous polymer composites via high internal emulsion templating. The hydrophilicity of Ca-BDC was measured through an aqueous phase by static contact-angle instrument. Results showed that Ca-BDC tablets have contact angles much less than 90° (25.5° ± 1, Figure S1B (top)), presenting relative hydrophilicity. The roles of different amounts of Ca-BDC in O/W HIPEs were initially determined according to the reported literatures [28] for testing the emulsifying ability of Ca-BDC particles. Cyclohexane-in-water HIPEs (O/W HIPEs) (internal volume fractions were 80%) solely stabilized by 1, 3, 5, and 7 wt % (relative to water) of Ca-BDC particles were formed (Figure 1A). Except for the HIPE emulsified by 1wt %, the other three HIPEs were inverted and kept stable for more than several months, and stability of these emulsions increase with the increasing amount of MOF particles dosage. To further investigate the appearances of sole Ca-BDC-emulsified O/W HIPEs, the aqueous phase was stained by a fluorescent indicator rhodamine B, the CLSM images and optical microscopy images (Figure S2) clearly show the nature of the big droplets (with cells size ranged from 50–300 μm) trapped by denser MOF layers. The emulsifying capability of Ca-BDC may mainly originate from the large excessive surface free energy caused by rest empty coordination. However, these O/W HIPEs containing small amounts of Ca-BDC possess uneven droplets and are easy to demulsify because of the disconnected Ca-BDC particles in CO_2_-water interfaces (Figure S2A,C).

**Scheme 1:**
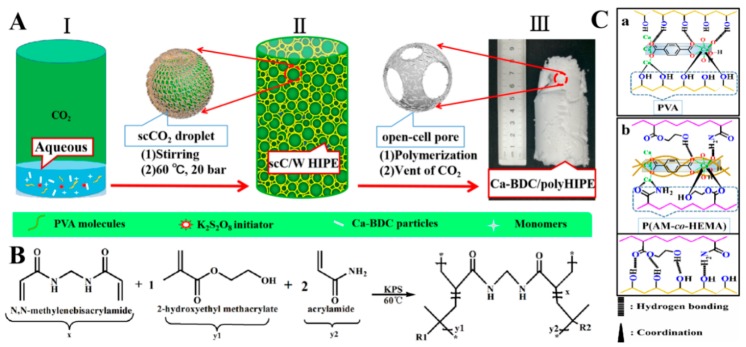
(**A**) Schematic illustration for the preparation of Ca-BDC/P(AM-*co*-HEMA)HIPEs; (**B**) typical radical polymerization of 2-hydroxyethyl methacrylate (HEMA), acrylamide (AM), and *N,N*’-methylenebisacrylamide (MBA) initiated by potassium persulfate (KPS) (x = n wt %, y1 + y2 =100 − n wt %) at 60 °C; (**C**) interactions between individual components in Ca-BDC/P(AM-*co*-HEMA) HIPEs.

According to the literatures reported [18,28], we added PVA molecules to interact with Ca-BDC to co-stabilized HIPEs. In detail, the hydroxyl groups of PVA can physically bond with the carboxyl groups on BDC linkers through hydrogen bonding, and combine the rest empty coordination orbitals of Ca^2+^ in Ca-BDC through coordination (shown in Scheme 1Ca). As a result, the resultingPVA modified Ca-BDC particles show more hydrophilicity (contact angle 18° ± 1 Figure S1B (bottom)). Next, utilization of this PVA modified Ca-BDC particles for emulsifying O/W HIPEs (co-stabilized O/W HIPEs) has been conducted to observe the changes of emulsion appearances and stability. It can be seen from the CLSM images (Figure 1E–G) that, after the addition of a little amount of PVA, more crowded droplets occurred compared to the Ca-BDC solely stabilized O/W HIPEs. With the increasing amount of Ca-BDC from 1 to 5 wt %, compared to PVA stabilized O/W HIPEs, the droplets sizes become slightly larger and more uniform (Figure 1D–G), resulting in more stable HIPEs formation (Figure 1B,C), which can be attributed to the improved viscosity of continuous phase in PVA co-stabilized HIPEs, and is the synergy of PVA and Ca-BDC. Finally, to verify whether the PVA-modified Ca-BDC particles possess the emulsifying ability for C/W HIPEs, after addition of a certain amount of PVA and Ca-BDC to co-stabilize C/W HIPE, followed by curing continuous phase, a white integral 3D PVA modified Ca-BDC macroporous monolith closely conformed to the vessel can be obtained (Figure 1H). Apparently, this monolith possesses clear macropores with lots of pore throats (Figure 1I,J). Moreover, the Ca-BDC particles were segregated into more fine nano-rods and networked tightly into the porous structure (Figure 1J,K), which further confirmed the stronger emulsifying ability of PVA-modified Ca-BDC for C/W HPEs (the mechanism of Ca-BDC and PVA-modified Ca-BDC emulsified C/W HIPEs formation are shown and elaborated in ESI). To the best of our knowledge, although Liu et al. [30] have first proposed the facile and green top-down approaches to the production of 3D hierarchical porous MOFs in MOFs emulsified CO_2_-in-H_2_O and multiple CO_2_-in-H_2_O-in-CO_2_ high internal phase emulsions, this is the first time that shaping MOFs into integral 3D macroporous monoliths was done in co-stabilize C/W HIPE. Ultimately, under the joint action of PVA molecules and MOF particles, robust protective barriers are formed among HIPEs droplets to effectively preventing the emulsion droplets from coalescence and demulsifying; therefore the HIPEs droplets are more uniform and stable. In view of the facile process as well as green and non-toxic components, these Ca-BDC emulsified C/W HIPEs have promising applications in food, cosmetic, pharmaceutical, as well as the production of MOFs-based monolith and MOF/PolyHIPEs composites.

### 3.2. Preparations of the Ca-BDC/PolyHIPEs Monolithic Composites

In practical applications, porous functional materials require not only specific functional ingredients but also a tough pore structure. However, it is clear that 3D porous PVA-modified MOF monolith does not possess sufficient mechanical strength; therefore its application is limited. As schematically showed in Scheme 1A,B, we take these Ca-BDC particles co-stabilized C/W HIPEs as templates to prepare Ca-BDC/polymer porous composites, to combine and perfect the structures and performances of 3D MOFs monoliths and polymer porous materials, through interactions between individual components. Simply, bio-compatible monomers HEMA, AM, cross-linker MBA, initiator KPS and Ca-BDC particles, PVA molecules were dispersed in the aqueous continuous phases, and a certain amount of CO_2_ was charged into the aqueous phases to be dispersed phases to form Ca-BDC co-stabilized C/W HIPEs containing soluble monomers. After radical polymerization of continuous phases at 60 °C, CO_2_ was transformed into supercritical CO_2_, which gave rise to the more stable C/W HIPEs. Followed by releasing CO_2_, white Ca-BDC/PolyHIPEs integral monolithic materials were obtained and named as Ca-BDC/P(AM-*co*-HEMA) HIPEs, which conform closely to the cylindrical interiors of the reaction vessels. Moreover, in these porous composite, the hydroxyl, amide, and ester groups of co-polymer P(AM-*co*-HEMA) can interact with MOFs Ca-BDC and PVA through hydrogen bondings and coordinations [28,34], which produce a robust Ca-BDC/polymer composite network (Scheme 1C). As controls, resulting polyHIPEs contain no monomer HEMA or Ca-BDC particles etc., were symbolized as Ca-BDC/P(AM)HIPEs, P(AM-*co*-HEMA)HIPEs, and so on, respectively. According to processes described above, a dozen porous polymer monoliths were prepared and abbreviated as PxCyWz, x, y, and z represent the content of Ca-BDC, CO_2_ and water in corresponding C/W HIPEs, respectively (see details in Table S1).

**Figure 2:**
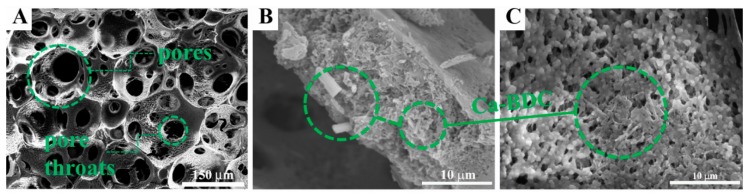
The pore structures and Ca-BDC distribution in (**A**) Ca-BDC/polyHIPEs: (**B**) and (**C**) are the cross-sectional view and surface of the pore of Ca-BDC/polyHIPEs, respectively, and Ca-BDC particles were marked with circles.

Based on the practical applications of biology, the different pore structures of polyHIPEs were desired, such as interconnected macropores with tunable size. To date, porous polymers templated from HIPEs, emulsified by traditional surfactants [18] and solid particles [19,21], have high porosity, but generally small pores and small pore throats or even closed-cell structure, which limited its applications in tissueengineering and drug delivery. Conventional technology to tune the average pore and pore throat size is employing additives [42,44] or a limited kinetic coarsening before polymerization [45], leading to complicated processes or toxic residues. In this work, the cavity remained after removal of CO_2_ droplets were defined as pores, and the “windows” to interconnect pores were defined as pore throats (Figure 2), which is attributed to the synergetic emulsification of PVA and Ca-BDC.

**Figure 3:**
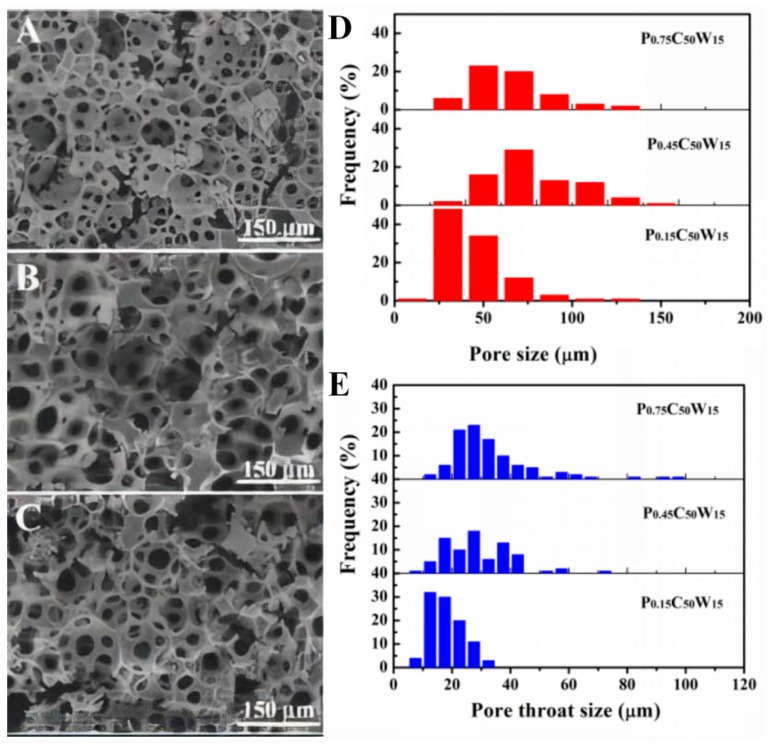
The SEM images (**A**), (**B**), and (**C**)of P**_x_**C**_50_**W**_15_** templated from C/W HIPEs containing 0.15, 0.45, and 0.75 g Ca-BDC, respectively; (**D**) and (**E**) are the distribution charts of pores and pore throats, respectively, which were measured and counted from corresponding SEM images.

In these Ca-BDC co-stabilized C/W HIPEs, Ca-BDC dosage, continuous phase volume, and CO_2_ pressure can affect the pore structures of resulting polyHIPEs. First, Figure 3 (left) shows the influence of the different amounts of Ca-BDC on the pore structures of these Ca-BDC/PolyHIPEs templated from co-stabilized C/W HIPEs (Table S1). Figure 3 (right) shows the distribution charts of the corresponding pores and pore throats. On the whole, with increasing amount of Ca-BDC from 0 to 0.75 g in corresponding C/W HIPEs, the pores get slightly large first and then more uniform (average pore size 38 μm of P_0.15_C_50_W_15_, 72 μm of P_0.45_C_50_W_15_ and 60 μm of P_0.75_C_50_W_15_). Besides, the pore throat sizes also increased and tend to be mono-dispersed, leading to a high level of pore interconnectivity (Table 1). These trends are similar to the works of O/W HIPEs (Figure 1) and can be explained as follows. On the one hand, it is the presence of PVA molecules that give rise to a separating film around the C/W HIPE droplets, which can effectively stabilize the C/W HIPEs [18]. On the other hand, the PVA molecules can modify the disconnected Ca-BDC particles through hydrogen bonds and coordination to form PVA-modified Ca-BDC. The rest of the PVA molecules act as binders to network the PVA modified Ca-BDC particles to co-stabilize these C/W HIPEs [28,46]. With an increasing amount of Ca-BDC particles, more PVA molecules in the continuous phase were absorbed onto the surface of Ca-BDC particles to produce more PVA modified Ca-BDC particles. It is the more PVA modified Ca-BDC particles that play the significant emulsifying role in P_0.45_C_50_W_15_and P_0.75_C_50_W_15_, resulting in large and more uniform pores than that of P_0.15_C_50_W_15_. While the amount of rest PVA in the continuous phase decreases, causing the thinner separating layer in the CO_2_-water interfaces and the larger open-cell structure than that ofP_0.15_C_50_W_15_ during curing of the continuous phase of C/W HIPE.

**Figure 4:**
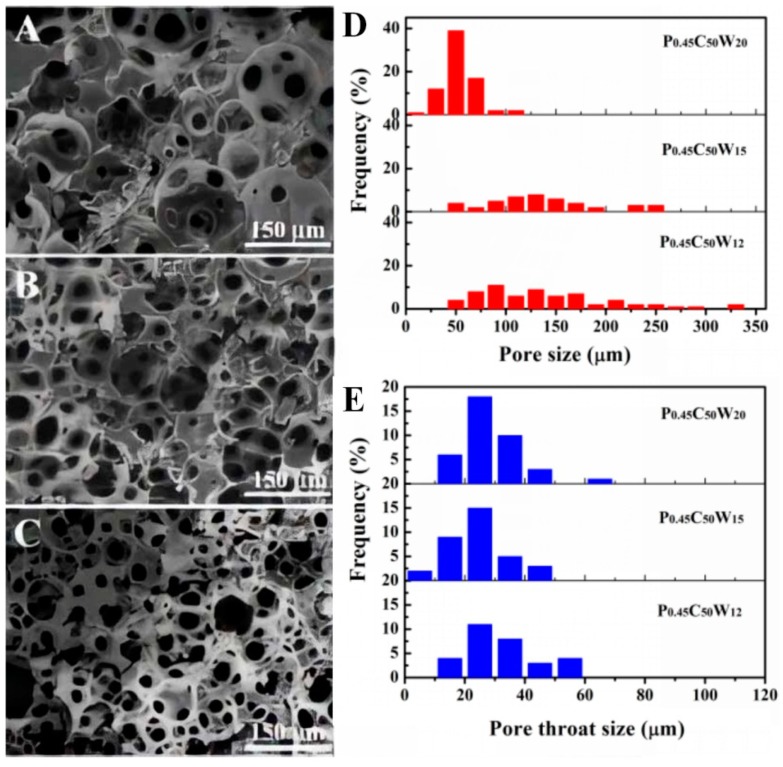
The SEM images of (**A**), (**B**), and (**C**)of P**_0.45_**C**_50_**W**_z_** templated from C/W HIPEs containing 12, 15, and 20 mL aqueous solution, respectively; (**D**) and (**E**) are the distribution charts of pores and pore throats, respectively.

Second, we adjust the pore morphologies of these polyHIPEs by altering the volume of the aqueous phase of C/W HIPEs. Excepting the P_0.45_C_50_W_12_, whose volume of monolith accounted for three-quarters of the reactor, the monoliths of P_0.45_C_50_W_15_ and P_0.45_C_50_W_20_ conformed closely to cylindrical interiors of the reaction vessels. As such, P_0.45_C_50_W_12_ (Figure 4A) possesses a little amount of pores with an average size of 115 μm and few pore throats. With further increasing the volume of deionized water in the C/W HIPEs, it is clear that the pore size and pore throat size decrease to become more uniform (Figure 4D,E), average pore size 125 μm for P_0.45_C_50_W_15_, decreased to 52 μm for P_0.45_C_50_W_20_, but the high level of pore connectivity is achieved. These results can be confirmed by a fact that, with an increasing volume of external continuous phase, the volume of the internal phase decreased, which results in reduced interfaces of CO_2_-water. Because of the ultra-low viscosity of CO_2_, under a fixed concentration of high-effective emulsifiers, emulsion droplets can be divided into more uniform and smaller crowded ones to generate more countered interfaces.

**Figure 5:**
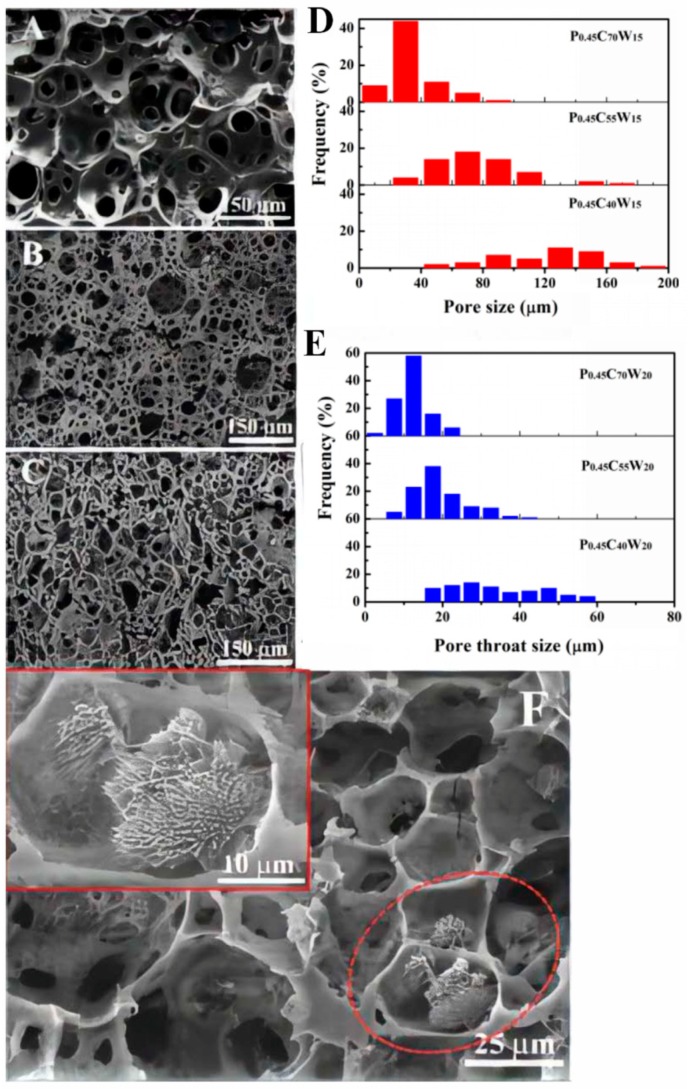
The SEM images of (**A**), (**B**), and (**C**) of P**_0.45_**C**_y_**W**_20_** templated from C/W HIPEs containing 40, 55, and70 g CO_2_, respectively; (**D**) and (**E**) are the distribution charts of pores and pore throats, respectively; (**F**) the secondary pore structure of P**_0.45_**C**_70_**W**_20_**templated from C/W HIPEs containing 70 g CO_2_.

Third, the pressures of CO_2_ in C/W HIPEs can exert significant influences on the pore morphologies of corresponding polyHIPEs. It can be observed in Figure 5, with an increasing amount of CO_2_, pore size and pore throat size decreased distinctly. Moreover, the pore size and pore throat size distribution got narrowed, respectively (Figure 5D,E). Compared to P_0.45_C_40_W_20_ and P_0.45_C_55_W_20_, both of which possess bigger pore with larger throats (average pore size 128 and 75 μm, respectively), and wide size distribution of pores and pore throats (see details in Table 1 and Figure 5A,B). The P_0.45_C_70_W_20_ has an irregular polyhedral pore structure with an average pore size of 27 μm and a pore throat size of 12 μm (Figure 5C). More interestingly, refined flower-like structures and rough surfaces were found in the pores of P_0.45_C_70_W_20_ (Figure 5F). Such a structure can increase the specific surface area, for example, which would provide this porous composite with the more active site for catalyst delivery. These effects on pore structure can be explained as, with increasing CO_2_ pressure of the system under fixed temperature, the polarity, density, dielectric constant, and salvation ability of CO_2_ increased [12,22], resulting in the decline of interface tension and free energy of the CO_2_-water interfaces. Therefore under unremitting stirring and a fixed concentration of emulsifiers, because of the ultra-low viscosity of CO_2_, more countered interfaces generated, and accordingly, a more stable HIPE with more crowed HIPE droplets or distorted cell structure can be formed.

### 3.3. Mechanical Performances

**Figure 6:**
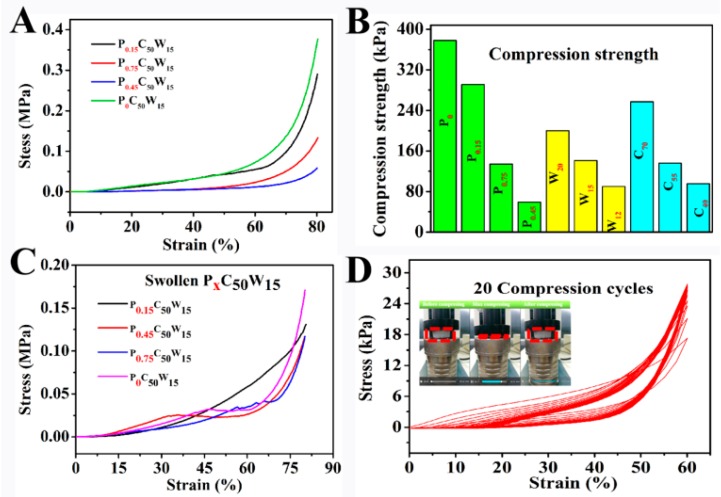
(**A**) Compression stress–strain curves of polyHIPEs P**_x_**C**_50_**W**_15_** templated from C/W HIPEs containing 0, 0.15, 0.45, and 0.75 g Ca-BDC respectively; (**B**) compression strengths of polyHIPEsP**_x_**C**_50_**W**_15_,**P**_0.45_**C**_50_**W**_x_**, and P**_0.45_**C**_x_**W**_20_** obtained from different amounts of Ca-BDC, water, and CO_2,_ respectively; (**C**) compression stress–strain curves of swollen polyHIPEs P**_x_**C**_50_**W**_15_** templated from C/W HIPEs containing 0, 0.15, 0.45, and 0.75 g Ca-BDC respectively; (**D**) the 20 compression cycles of polyHIPEs P**_0.45_**C**_70_**W**_20_**.

Commonly, polyHIPEs porous materials are designed necessarily to offer adequate mechanical support according to practical applications. The typical performances of the polyHIPEs (tested as freshly obtained, i.e., 3 g specimens contains about 2 g H_2_O) in the three processes of compression test are shown in Figure 6 (insert) and the Video. All of polyHIPEs exhibiting excellent compression resistance can regain their original size after removal of compression stress. Corresponding compression strain–stress curves were plotted in Figure 6A and Figure S3. All of the curves consist of three linear regions with increasing positive slopes, which are characteristic of open-cell flexible polymer foams [44]. When the strains reach 80%, most compression strengths of as-prepared polyHIPEs are within a range of 70 kPa to 319 kPa (Figure 6B). Additionally, swollen porous composites of P_x_C_50_W_15_ with water were also carried out to test compression strain–stress behavior. Results came out that these polymer composites also have partial mechanical strength (Figure 6C), which can endow them certain mechanical support for applications of bio-tissue engineering and drug delivery.

Moreover, mechanical strength enhanced with decreased pore size (Figure 6B and Figure S3A,B), which is attributed to the fact that the more uniform and smaller pore can provide a porous matrix with more supporting points. However, with the increasing content of Ca-BDC, these porous composites became more flexible (Figure 6A). This trend caused by: (1) The introduction of Ca-BDC into these polyHIPEs decreased the amount of hydrogen bonds among PVA and P(HEMA-*co*-AM) molecules; (2) the bigger pores with larger pore throats of these Ca-BDC/polyHIPEs lead to the softness of composites and more deformation capacity of the pores. Cyclic compression of P_0.45_C_70_W_20_ was conducted at a strain range of 0–60%, as we can see, after 20 cycles of compression, the profiles of corresponding stress–strain curves can be roughly maintained the same as it was initially tested (Figure 6D), indicating a superior elasticity to prevent this porous composites from fracture in practical bio-applications. In addition, the obvious hysteresis of the porous composite means that energy dissipation existed. This phenomenon may be resulted from that the porous structure of the composite can be reversibly deformed and physical interaction among polymers and Ca-BDC. It can reversibly associate and disassociate to dissipate stress effectively.

### 3.4. General Characterization

**Figure 7:**
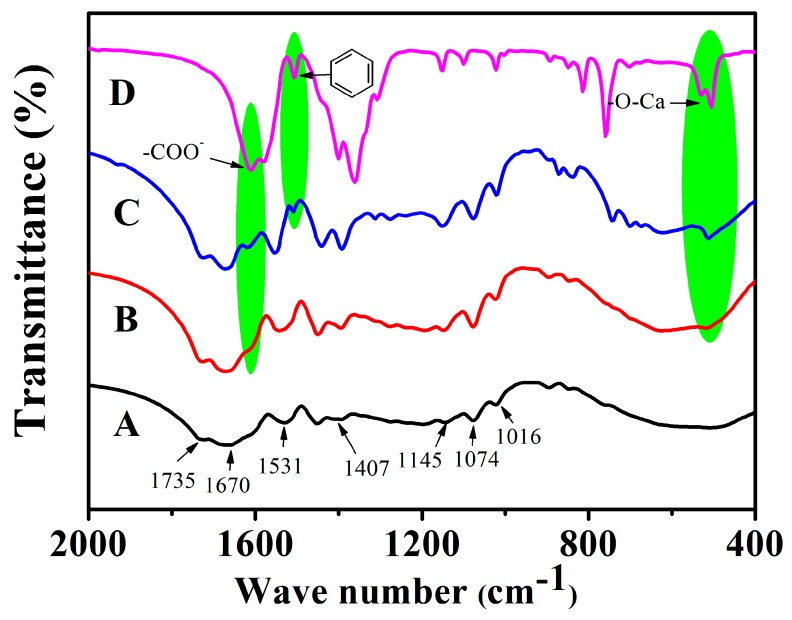
The FT-IR spectra of (**A**) P**_0_**C**_50_**W**_15_**, (**B**) P**_0.75_**C**_50_**W**_15_**, and (**C**) P**_3_**C**_50_**W**_15_**, (**D**) as-synthesized Ca-BDC.

Fourier transform infrared spectroscopy first determined the chemical compositions of these Ca-BDC/polyHIPEs. Infrared spectra of as-synthesized Ca-BDC, P**_0_**C**_50_**W**_15_** as well as composites P**_x_**C**_50_**W**_15_** containing different amount of Ca-BDC are shown in Figure 7. In details, the characteristic bands centered at 1670 and 1407 cm^−1^ belong to strong carbonyl stretching vibration and C–N stretching vibration of amide groups of PAM, respectively. The strong peaks at 1735 and 1145 cm^−1^ are assigned to carbonyl stretching vibration and C–O–C stretching vibration of ester groups of PHEMA. In the spectra of P**_x_**C**_50_**W**_15_**, it can be observed that the peaks at 1670, 1735, and 1145 cm^−1^ in spectra of co-polymer P**_0_**C**_50_**W**_15_** also exist in these polymer composites. Besides, with increasing amount of Ca-BDC in these composite, some new peaks at 1612, 1509, and 506 cm^−1^ occurred and become stronger in co-polymer, which belong to the asymmetric stretching vibrations of deprotonated carboxyl groups, benzene ring skeleton, and calcium-oxygen bonds in Ca-BDC, respectively.

**Figure 8:**
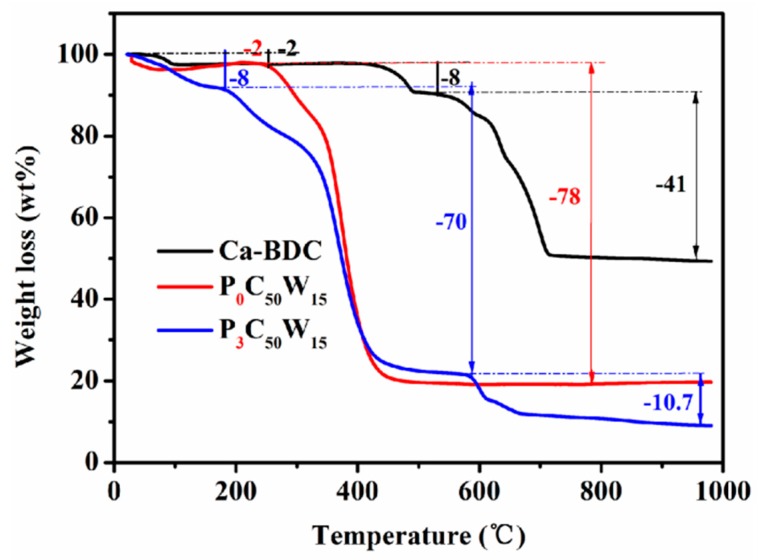
The TG curves of Ca-BDC, P**_0_**C**_50_**W**_15_** and P**_3_**C**_50_**W**_15_** templated from C/W HIPEs containing 0 and 3 g Ca-BDC, respectively.

TG analysis of P**_0_**C**_50_**W**_15_** containing no Ca-BDC, P**_3_**C**_50_**W**_15_** containing 3 g Ca-BDC, and as-synthesized Ca-BDC in a flow of nitrogen with a heating rate of 10 °C/min are shown in Figure 8. The TG curve of as-synthesized Ca-BDC possesses three obvious weight-loss stages, which is consistent with the reported literature [47]. The initial 2% weight-loss between 0–100 °C is caused by the loss of physically adsorbed water molecules. The second 8% weight-loss between 200 and 500 °C is mainly attributed to the removal of coordinated water and DMF molecules. The third 41% weight-loss is a result of the decomposition of the Ca-BDC framework. The TG curve of P**_0_**C**_50_**W**_15_** containing no Ca-BDC have mainly two thermal degradation stages. The initial 2% degradation before 100 °C resulted from the loss of physically adsorbed water. The second 78% degradation between 200 and 450 °C is caused by the decomposition of polymer chain. The TG curve of P**_3_**C**_50_**W**_15_** also consists of three degradation stages; the first 8% weight-loss before 200 °C resulted from the loss of physically adsorbed water molecules. The decomposition of polymer chains cause the second 70% degradation between 200 and 600 °C; the third 10.7% weight-loss may be the result of the decomposition of Ca-BDC framework, which is expected −12%, calculated from eq: ($\frac{-M_{\left(\mathrm{Ca}-\mathrm{BDC}600\right)}\times 41\%}{M_{\left(\mathrm{Ca}-\mathrm{BDC}600+\mathrm{Polymers}\right)}})\times 100\%$, (Ca-BDC (Ca-BDC_600_) contains no water and DMF at 600 °C [48], presumed that the finally remained phase is CaCO_3_, the weight-loss of Ca-BDC_600_ is 41% beyond 600 °C). Differently, the weight-loss between 200 and 500 °C attributed to the removal of coordinated water and DMF molecules disappeared, indicating Ca-BDC contained in these polymer composites does not involve DMF and water any more. Therefore there may be a restructuring process in C/W HIPEs, in which CO_2_ extracted the DMF molecules through a structural transformation of Ca-BDC [48]. This phenomenon was also found in Liu’s reported work [30]. Structural changes among Ca(BDC)(DMF)(H_2_O) and Ca(BDC)(H_2_O)_3_ can easily occur under relatively moderate conditions [41,47] (see more details in ESI).

**Figure 9:**
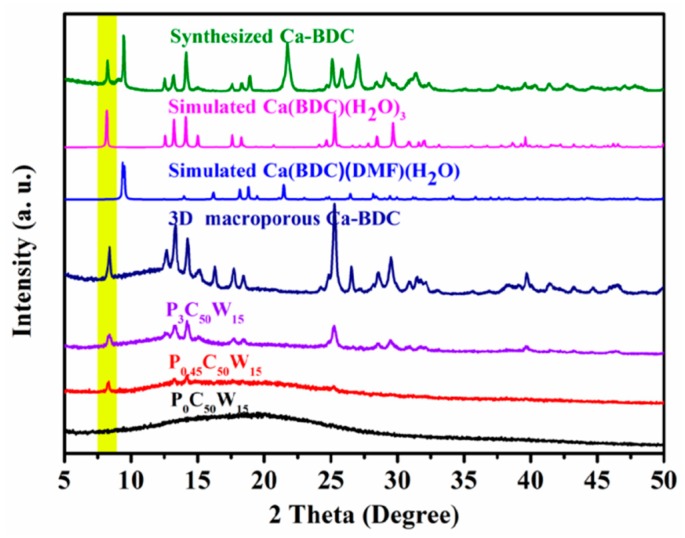
The XRD patterns of polyHIPEs P**_x_**C**_50_**W**_15_** templated from C/W HIPEs containing 0, 0.45, and 3 g Ca-BDC, respectively, and 3D macroporous Ca-BDC.

The crystal phase identification of pure as-synthesized Ca-BDC and the as-prepared polyHIPEs were conducted by powder XRD. As shown in Figure 9, as-synthesized Ca-BDC possesses two typical intense diffraction peaks at 2θ = 5°–10°, while the typical intense diffraction peaks of 3D macroporous Ca-BDC integral monolith and Ca-BDC/PolyHIPEs match well with those of pure simulated Ca(BDC)(H_2_O)_3_ [41], which have only one intense diffraction peaks at 8.2°, indicating the presence of highly crystallized Ca(BDC)(H_2_O)_3_ in Ca-BDC/PolyHIPEs composites and 3D macroporous Ca-BDC monolith. These results further confirmed that there exists a restructuring process for Ca-BDC in C/W emulsion [45]. All of TG and PXRD results above suggest that the successful embedding of Ca-BDC particles into these polyHIPEs, while toxic DMF was removed in C/W HIPEs, is beneficial for Ca-BDC to be applied in bio-fields.

**Figure 10:**
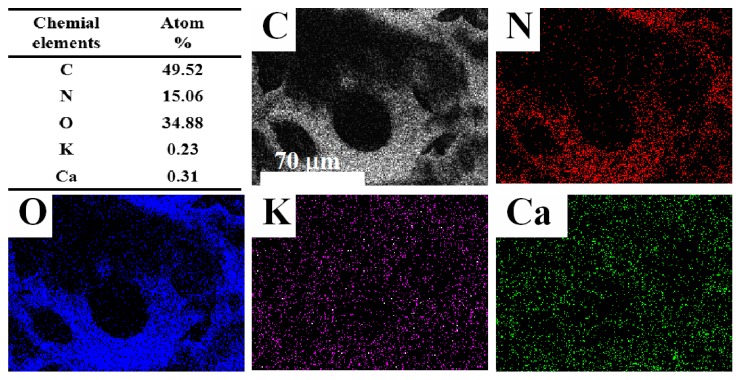
The mapping images and EDS chart of Ca-BDC/P(AM-*co*-HEMA)HIPE composite.

The surface chemical compositions of porous material are crucial for its application. Mapping images of the pore surface of Ca-BDC/PolyHIPE were obtained to determine the distribution of every element. As shown in Figure 10, excepting the C, N, K, and O, Ca element is distributed uniformly on the composite pore surface. These results are also in agreement with the SEM images in Figure 2, where the Ca-BDC particles were well-dispersed on the surfaces of the pores or embedded into the pores wall, demonstrating that Ca-BDC was dispersed uniformly into Ca-BDC/polyHIPE, which is critical for Ca-BDC to behave their bio-functions, and partially proved the emulsifying role of the Ca-BDC particles.

### 3.5. Bio-Compatibility Evaluations

**Figure 11:**
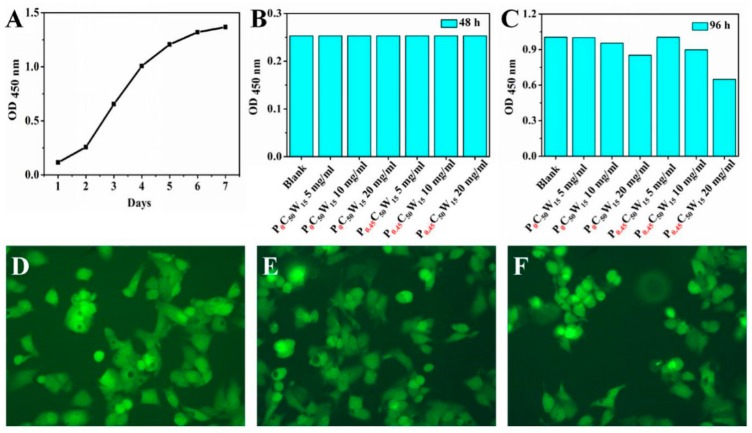
(**A**) Standard growth curves of HpEG2 cells; (**B**) and (**C**) are the effects of different dosages of P**_0_**C**_50_**W**_15_** and P**_0.45_**C**_50_**W**_15_**on HepG2 cells proliferation for 48 h and 96 h respectively; (**D**) the fluorescent micrographs (300×) of HepG2 cell grown under normal condition; (**E**) and (**F**) are fluorescent micrographs of HepG2 cell grown for 48 h with presence of P**_0_**C**_50_**W**_15_** and P**_0.45_**C**_50_**W**_15_** under low dosage (5 mg/mL).

Bio-compatibility is a critical requirement for bio-materials [49]. Effects of as-synthesized Ca-BDC/P(AM-*co*-HEMA)HIPEs on HepG2cell proliferation are shown in Figure 11. After co-culturing with P_0_C_50_W_15_ and P_0.45_C_50_W_15_ under different dosages for 48 h, the HepG2 cells’ growths of all groups were not inhibited at all and with good growth states (Figure 11B,E,F). After further co-culturing for 96 h, the OD values of cell proliferation slightly declined, but with a good growth state. Additionally, compared to P_0_C_50_W_15_ without Ca-BDC, P_0.45_C_50_W_15_ templated from C/W HIPEs containing 0.45 g Ca-BDC show lower OD values of cell suspensions (Figure 11C), which may be attributed to that more cell grew into the larger pores of P_0.45_C_50_W_15_. These results show that the size of the pores and Ca-BDC contents in these Ca-BDC/P(AM-*co*-HEMA) HIPEs materials are vital for cell proliferation (the OD values and micrographs of HepG2cells proliferation in all groups are summarized in Table S1 and Table S2).

**Figure 12:**
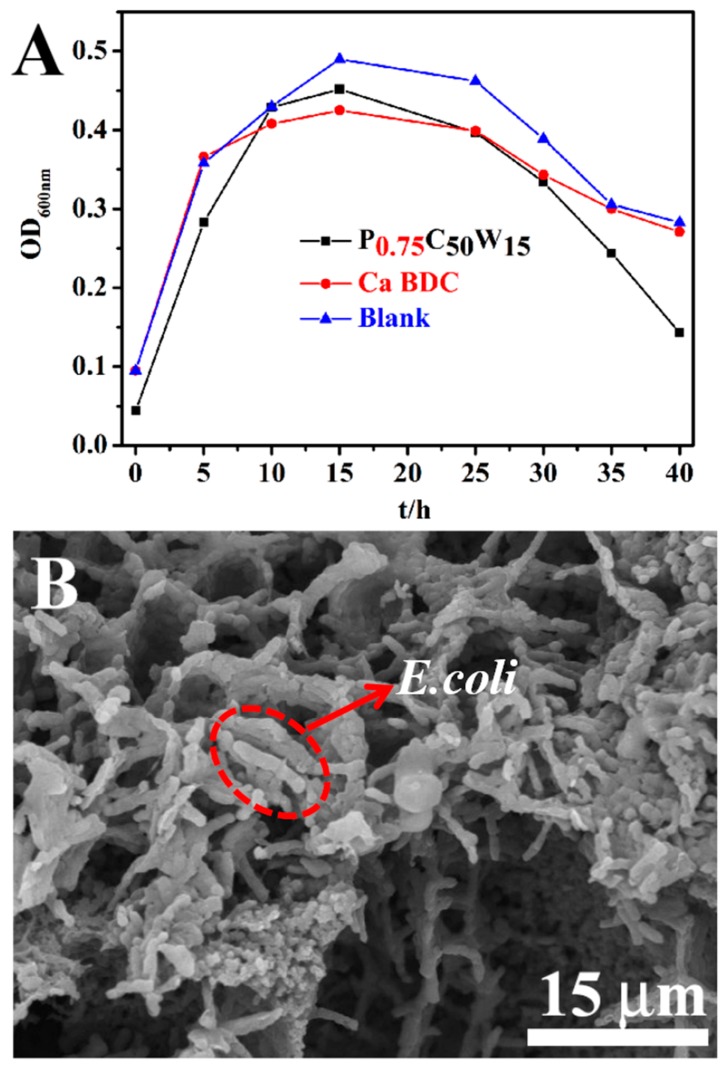
(**A**) Growth curves of *E. coli* under normal conditions and as-synthesized Ca-BDC and P_0.75_C_50_W_15_ exist; (**B**) the SEM images of *E. coli* grown in P_0.75_C_50_W_15_.

To further determine the bio-compatibility, *E. coli* cells were grown under the presence of as-prepared P_0.75_C_50_W_15_ and Ca-BDC particles. The OD values of *E. coli* grew during 0–40 h were measured at fixed intervals and are plotted in Figure 12A (recorded in Table S3), and the cells’ growth in P_0.75_C_50_W_15_ containing 0.75 g Ca-BDC was captured in SEM image of Figure 12B. It can be seen from Figure 12A that the growth of free *E. coli* shows four parts: stagnation, logarithmic growth phase, stationary phase, and decay phase. The growth curves of *E. coli* in the presence of P_0.75_C_50_W_15_ and Ca-BDC particles have a similar trend compared to the free one. Differently, in their initial stages, the OD values of *E. coli* grown under the presence of P_0.75_C_50_W_15_, was slightly lower than that of the free one, probably it can be attributed to that the porous polymer containing Ca-BDC can absorb *E. coli* into the pores of P_0.75_C_50_W_15_. Also, the SEM images (Figure 12B) show that *E. coli* cannot only live on the surfaces but also grow in the pores of P_0.75_C_50_W_15_. It indicated that P_0.75_C_50_W_15_ and Ca-BDC had no obvious inhibition on the growth of *E. coli*, and it also reflected that these Ca-BDC/P(AM-*co*-HEMA) HIPEs have good bio-compatibility. Note that favorable bio-compatibility of these polyHIPEs can be attributed to non-toxic solvents contained in C/W HIPEs, bio-compatible copolymer P(AM-*co*-HEMA), and bio-active MOFs Ca-BDC.

### 3.6. Evaluation of β-amylases Immobilization

**Figure 13:**
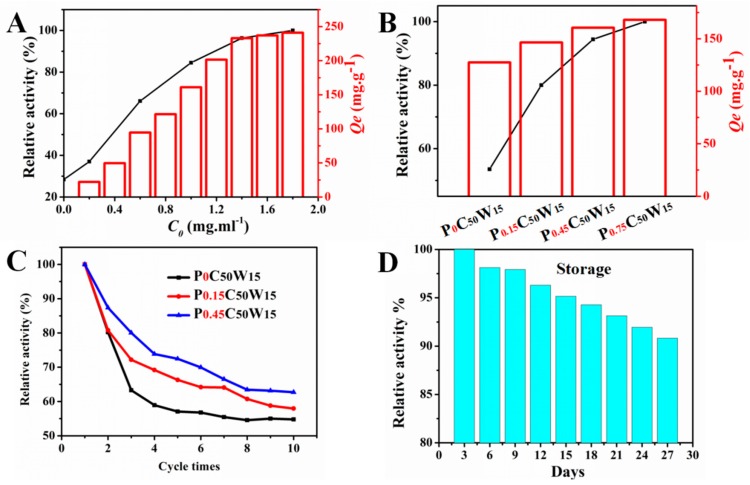
(**A**) Immobilizing *β*-amylasesintoP_0.75_C_50_W_15_conducted under enzyme solutions (pH = 6) with different enzyme concentration; (**B**) under enzyme concentration of 1.0 mg/mL (pH = 6), immobilizing *β*-amylases into P_X_C_50_W_15_ and P_0_C_50_W_15_; (**C**) the reusability of immobilized *β*-amylases prepared by P_0.15_C_50_W_15_, P_0.45_C_50_W_15_ and P_0_C_50_W_15_ under enzyme solution (1.4 mg/mL) with pH = 5; (**D**) the storage stability of immobilized *β*-amylases prepared by P_0.75_C_50_W_15_ under enzyme solution (1.4 mg/mL) with pH = 5.

Various enzymes play vital roles in cell proliferation, differentiation, and metabolism. But the enzyme activity can be easily affected by environments. Owing to the presence of Ca-BDC, amino, and hydroxyl groups in these Ca-BDC/P(AM-*co*-HEMA)HIPEs, *β*-amylases can be immobilized through chemical cross-linking [47] or adsorption [48] into as-synthesized Ca-BDC/P(AM-*co*-HEMA) HIPEs. Given the complicated processes and hazardous residues of chemical cross-linking in resulting products, we choose adsorption to immobilize *β-*amylases byphysical bonding. These Ca-BDC/polymer composites possess excellent adsorption capacity for *β*-amylase, while the enzyme activity can be highly maintained. It can be seen from Figure 13 that immobilization of *β*-amylases were carried out under different *β*-amylase concentrations of 0–2 mg/mL and utilization of P_x_C_50_W_15_ containing different amount of Ca-BDC. Next, the resulting immobilized *β*-amylases were employed to test their catalytic performance in starch solutions. Results came out that, with increasing initial enzyme concentration and increasing Ca-BDC content in P_x_C_50_W_15_, the adsorption capacity showed an upward trend, and came to be stable up to 250 mg/g of P_0.75_C_50_W_15_ at *β*-amylase concentration of 2 mg/mL. At the same time, enzyme activity was improved simultaneously. These immobilized *β*-amylases also display superior endurability. After reusing the immobilized *β*-amylases ten times, the enzyme activity of immobilized *β*-amylases prepared by P_0.45_C_50_W_15_ can maintain about 65% of their initial activity. After storage of the immobilized *β*-amylases prepared by P_0.45_C_50_W_15_ for 27 days, their catalytic activity can maintain 90% of that of freshly prepared. These positive results may be mainly caused by the rich hydroxyl, carbonyl, and amine groups, as well as the increasing amount of bio-active Ca-BDC in these porous composites, which increase *β*-amylase adsorption via hydrogen bonds and coordination. Meanwhile, the optimal conformation of the enzyme molecules were maintained by calcium of Ca-BDC [49].

## 4. Conclusions

In summary, this work developed a green and effective bio-active Ca-BDC co-stabilized C/W HIPE, in which CO_2_ was the dispersed phase, and PVA aqueous phase containing Ca-BDC particles as co-stabilizers is the continuous phase. PVA molecules in continuous phase can modify Ca-BDC particles, and network Ca-BDC particles into separating films, thus generating the more uniform HIPE droplets and more robust C/W HIPEs. Based on these co-stabilized C/W HIPEs, after freezing and decompressing, a kind of integral 3D Ca-BDC macroporous monolith material was produced. As a result, a series of Ca-BDC/P(AM*-co*-HEMA)HIPEs monolithic composites with well-defined tunable open-cell macro-porous structure were successfully prepared through polymerization of continuous phases of these Ca-BDC co-stabilized C/W HIPEs containing soluble monomers. Next, through tailoring formulas of these C/W HIPEs, the properties (pore structures and mechanical performances) of corresponding polyHIPEs can be tailored. Especially, CO_2_ pressure can exert a significant influence on the pore structure, refined flower-like and distorted pore structures were found in Ca-BDC/PolyHIPEs templated from C/W HIPE containing much more CO_2_, which enable the porous material large surface area for catalyst delivery, etc.

Furthermore, we found that rod-like Ca-BDC particles, well-dispersed onto and embedded in the surface of the polymer pores, play roles in providing these porous composites with specific bio-activity. Accordingly, the Ca-BDC/PolyHIPEs show reasonable bio-compatibility. In essence, these Ca-BDC/PolyHIPEs showed no significant inhibition on the growth of HepG2 cells and *E. coli*, indicating non-cytotoxicity of these Ca-BDC/P(AM-*co*-HEMA)HIPEs. The successful immobilization of *β*-amylases into this kind of materials through physical adsorption presents that, Ca-BDC/PolyHIPEs cannot only physically bond with the enzyme tightly but also maintain its optimal conformation to improve their catalytic activity. Furthermore, this green method and novel material can be applied in biological and food fields, such as tissue engineering and drug delivery.

## Figures and Tables

**Table 1:** Micro-morphological characteristics of different Ca-BDC/polyHIPEs were measured and counted from corresponding SEM images.

Samples	PxC_50_W_15_^a^			P_0.45_C_50_Wy ^b^			P_0.45_C_z_W_15_^c^		
Variable	0.15	0.45	0.75	12	15	20	40	55	70
d/D (%)	47	36	47	33	29	42	23	22	44
d (μm)	18	26	28	38	36	22	30	17	12
D (μm)	38	72	60	115	125	52	128	75	27

^a^ P**_x_**C**_50_**W**_15_** templated from C/WHIPEs containing different amount (g) of Ca-BDC. ^b^ P**_0.45_**C**_50_**W**_y_**templated from C/WHIPEs containing different amount (mL) of deionized water. ^c^ P**_0.45_**C**_z_**W**_15_** templated from C/WHIPEs containing different amount (g) of CO_2_. **D** represents average pore size, **d** represents average pore throats size, **d/D** represents the level of pore connectivity.

## Supplementary Materials

The following are available online at https://www.mdpi.com/2073-4360/12/4/931/s1, Scheme S1: The synthesis of (**a**) Ca(BDC)(DMF)(H_2_O) and (**b**) Ca(BDC)(H_2_O)_3_. Figure S1: (**A**)The SEM image of as-synthesized Ca-BDC crystals; (**B**) contact angles of as-synthesized Ca-BDC tablet (top) and PVA modified Ca-BDC tablet (bottom); (**C**) the FT-IR spectra of as-synthesized Ca-BDC and H_2_BDC; (**D**) the PXRD patterns of Ca-BDC-DMF-H_2_O, Ca-BDC-H_2_O and as-synthesized Ca-BDC. Figure S2: (**A**) Solely stabilized O/W HIPEs (1), (2), (3), and (4) containing 1, 3, 5, and 7 wt % of Ca-BDC to water phase, respectively; (**B**) and (**C**) are the CLSM images of O/W HIPE (1) and (3); (**E**) and (**F**) are the SEM images of the microstructure of sole Ca-BDC monolith obtained from sole Ca-BDC emulsified O/W HIPE. Scheme S2: The formation of Ca-BDC and PVA modified Ca-BDC particles emulsified C/W HIPEs and the preparation of 3D Ca-BDC MOFs monoliths. Figure S3: (**A**), (**B**) Compression stress–strain curves of P_0.45_C_50_W_X_ and P_0.45_C_x_W_20_ templated from C/W HIPEs containing different water and CO_2_, respectively. Figure S4: (**A**) Immobilizing *β*-amylases into P_0.75_C_50_W_15_ conducted under enzyme solutions with different pH; (**B**) *β*-amylases and immobilized ones (prepared by P_0.75_C_50_W_15_ in enzyme solution (1.4 mg/mL) with pH = 5) work in starch solutions at different temperature (pH = 5); (**C**) free *β*-amylases and immobilized ones (prepared by P0.75C50W15 in enzyme solution (1.4 mg/mL) with pH = 5) work in starch solutions with different pH (50 °C). Table S1: The formulas of C/W HIPEs. Table S2: The micrographs of HepG2 cell proliferation in all groups (200×). Table S3: The OD450 nm values of HepG2 cell proliferation in all groups. Table S4: The OD600nm values of E. coli proliferation in all groups.
