# Supplementary material for: Bio-Compatible Ca-BDC/Polymer Monolithic Composites Templated from Bio-Active Ca-BDC Co-Stabilized CO2-in-Water High Internal Phase Emulsions
Source: Polymers (Basel). 2020 Apr 17;12(4):931. doi: 10.3390/polym12040931 (PMC7240421; doi:10.3390/polym12040931)
Supplement: Supplementary file 1 [file polymers-12-00931-s001.zip › supplementary materials/RE polymers-753165-supplementary.docx]

Supplementary Materials:

Bio-compatible Ca-BDC/Polymer Monolithic Composites Templated from Bio-active Ca-BDC Co-Stabilized CO_2_-in-Water High Internal Phase Emulsions

Xule Yang^1^, Youwei Hao^1^ and Liqin Cao^1^*

^1^ Key Laboratory of Oil and Gas Fine Chemicals, Ministry of Education & Xinjiang Uygur Autonomous Region, Xinjiang University, Urumqi, 830046 P.R. China


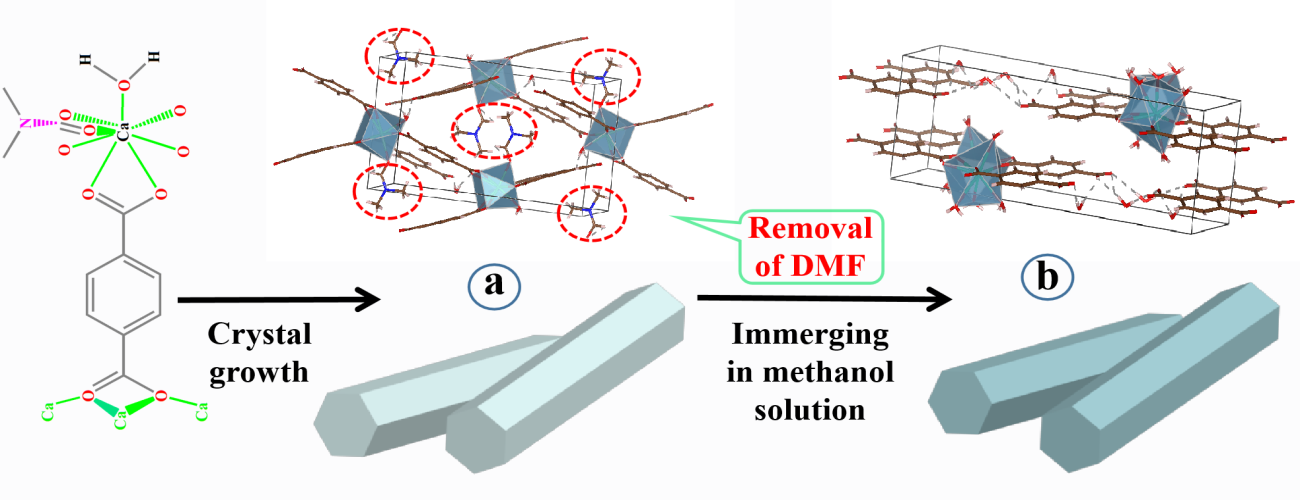


**Scheme S1.** The synthesis of (**a**) Ca(BDC)(DMF)(H_2_O) and (**b**) Ca(BDC)(H_2_O)_3_


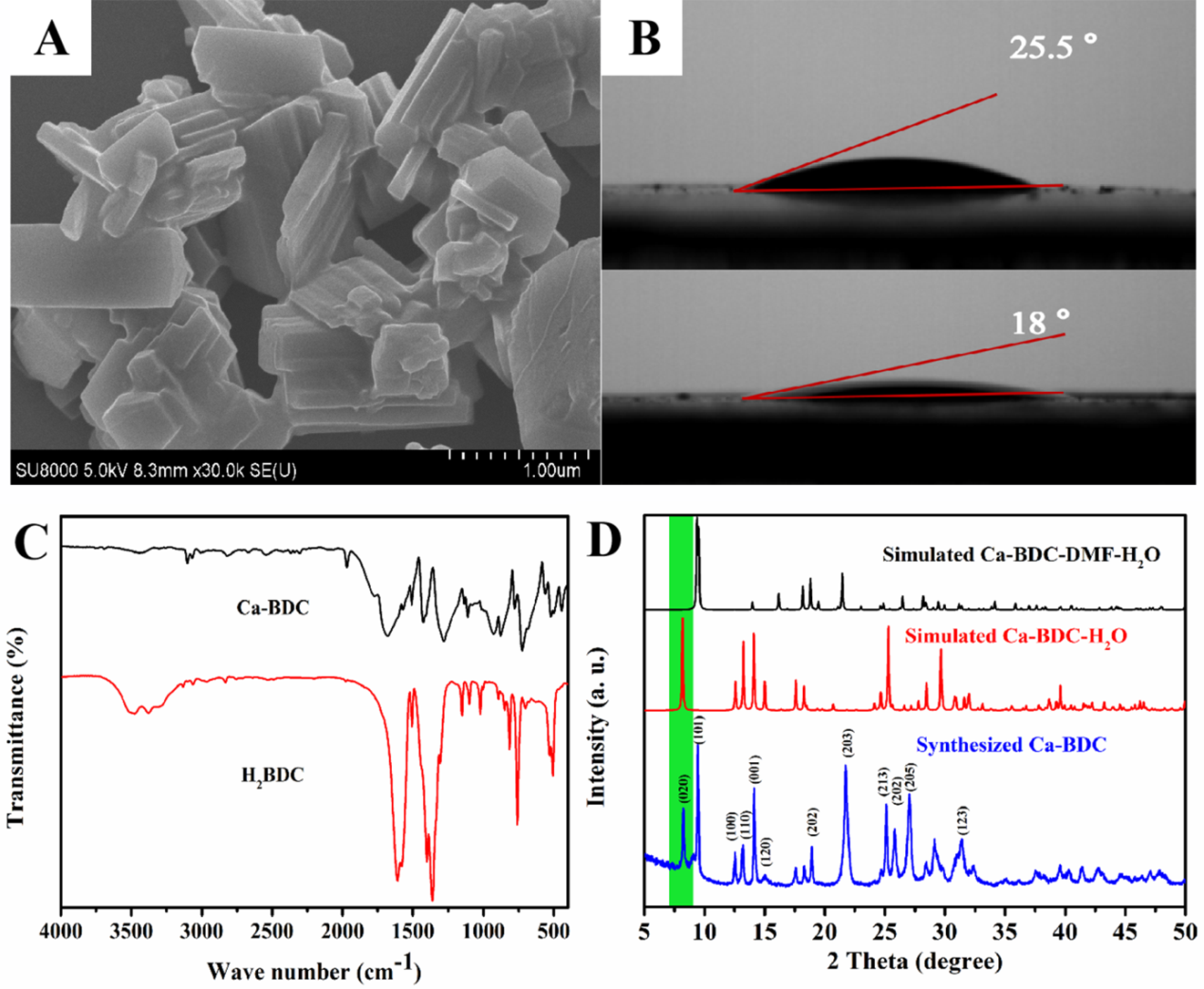


**Figure S1.** (A)The SEM image of as-synthesized Ca-BDC crystals; (B) Contact angles of as-synthesized Ca-BDC tablet (top) and PVA modified Ca-BDC tablet (bottom); (C) The FT-IR spectra ofas-synthesized Ca-BDC and H_2_BDC; (D) The PXRD patterns of Ca-BDC-DMF-H_2_O,Ca-BDC-H_2_O andas-synthesized Ca-BDC.

It is reported that there is a structural changes among Ca(BDC)(DMF)(H_2_O) and Ca(BDC)(H_2_O)_3_ under relatively moderate condition[[1](#_ENREF_1), [2](#_ENREF_2)], in which the toxic DMF molecules can be removed.

[Ca(BDC)(DMF)(H_2_O)] MeOH/H_2_O [Ca(BDC)(H_2_O)_3_]

Herein we synthesized a Ca-BDC MOF, which is a hybrid of Ca(BDC)(DMF)(H_2_O) and Ca(BDC)(H_2_O)_3_.The SEM image (**Figure S1A**) shows that the as-synthesized MOFs are agglomerates comprised of polyhedral rod-like crystals with width of 50 nm and length of several microns. Infrared spectra of as-synthesized Ca-BDC are shown in **Figure S1C** and are consistent with other literature data[[1](#_ENREF_1), [2](#_ENREF_2)]. The strong bands at 1600 and 1400 cm^-1^ are assigned to the asymmetric and symmetric vibrations of the carbonyl group, respectively. The absence of a band in the range between 1680 and 1800 cm^-1^ indicates the presence of only deprotonated carboxyl groups. The band at 1657 cm^-1^ corresponds to the C=O vibrations of the DMF molecule. Powder X-ray diffraction pattern (**Figure S1D**) of as-synthesized MOF are a superposition of individual pattern of both MOFs Ca(BDC)(DMF)(H_2_O) and Ca(BDC)(H_2_O)_3_[[2](#_ENREF_2)].Especially, the peaks respectively assigned to crystal planes of (020) of Ca(BDC)(H_2_O)_3_ and (101) of Ca(BDC)(DMF)(H_2_O) exist in as-synthesized Ca-BDC, which well proved that the products are the hybrids of MOFs Ca(BDC)(DMF)(H_2_O) and MOF Ca(BDC)(H_2_O)_3_.


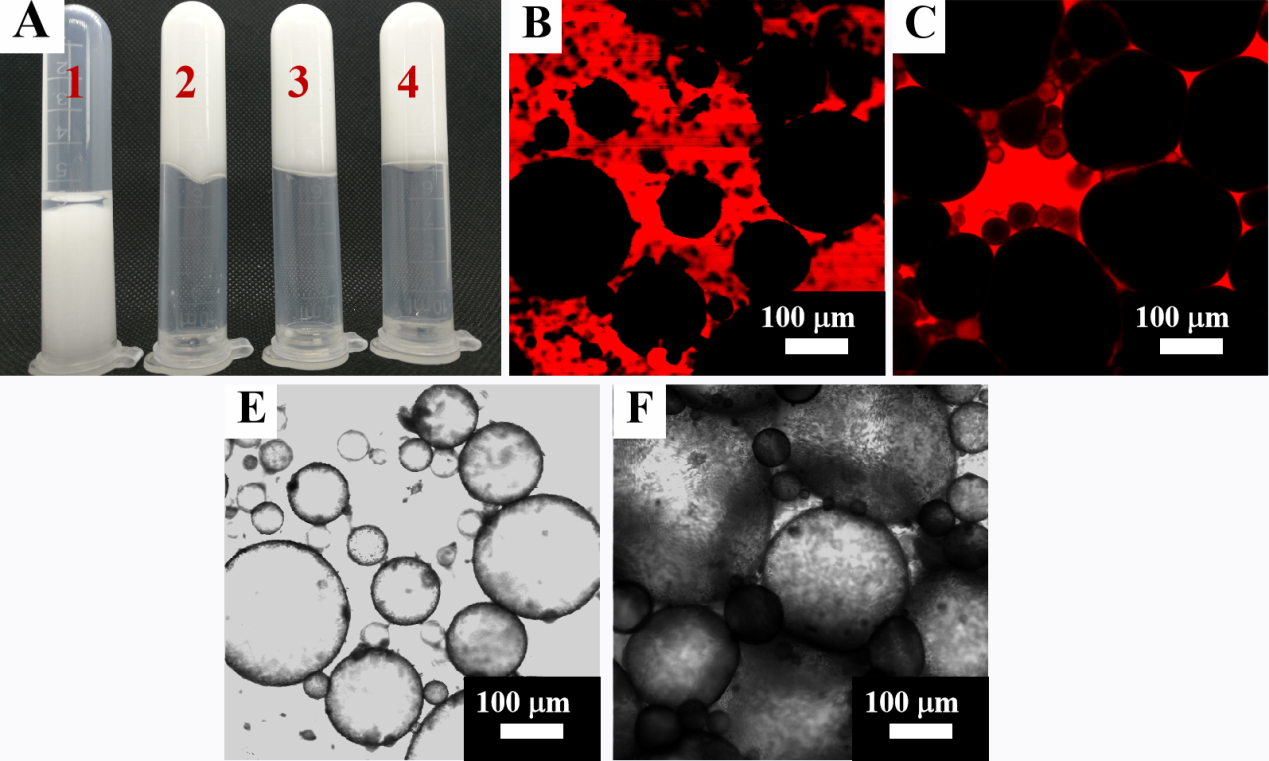


**Figure S2.** (A) solely stabilized O/W HIPEs (1), (2), (3), and (4) containing 1, 3, 5 and 7wt% of Ca-BDC to water phase, respectively; (B) and (C) are the CLSM images of O/W HIPE (1) and (3); (E) and (F) are the SEM images of the microstructure of sole Ca-BDC monolith obtained from sole Ca-BDC emulsified O/W HIPE.


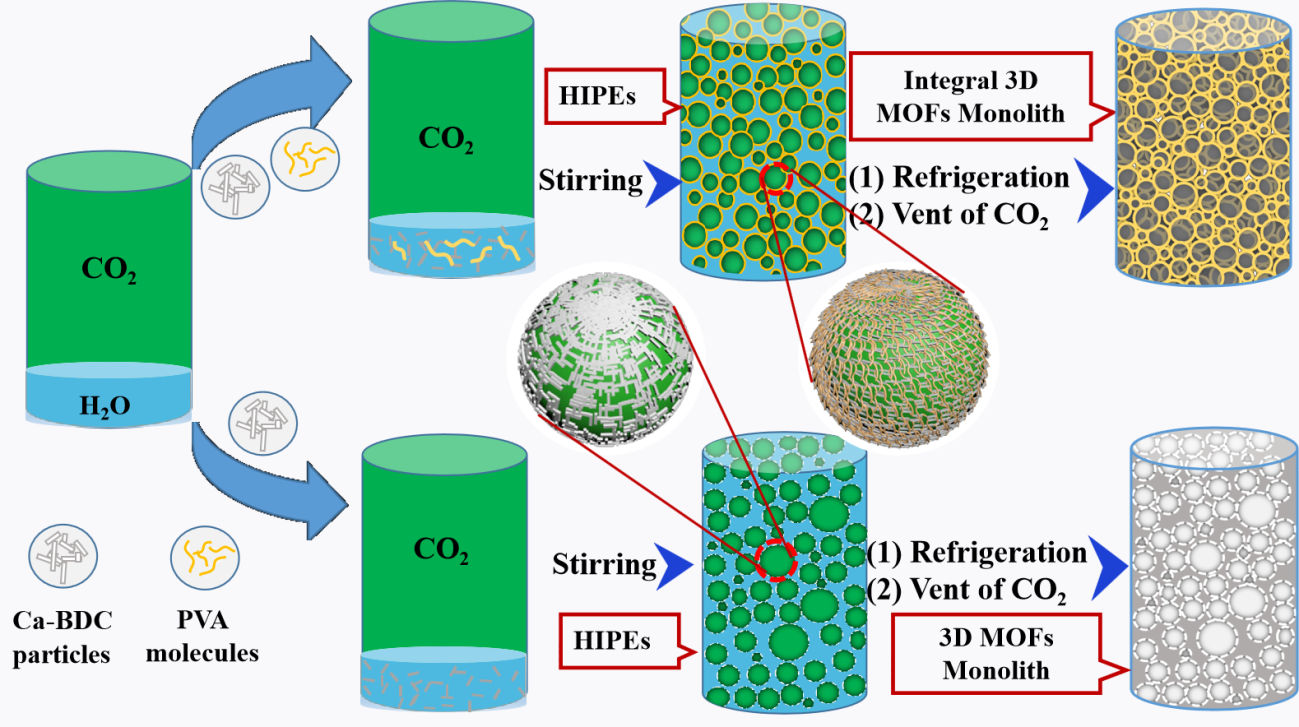


**Scheme S2.** The formation of Ca-BDC and PVA modified Ca-BDC particles emulsified C/W HIPEs and the preparation of 3D Ca-BDC MOFs monoliths.

The mechanism of MOFs solely emulsified C/W HIPEs formation was explained in reported literatures[[3](#_ENREF_3)] and schematically shown in **Scheme S2**. Due to the large surface excessive free energy of statically charged Ca-BDC particles (negative zeta potential of -12.5 mV in water at room temperature), MOF particles can be irreversibly absorbed or self-assembled on the CO_2_-water interfaces to form denser rigid separating layers, which can effectively counter the CO_2_-water interfaces free energy. And it is that under the continuous energy input, MOF particles were segregated into ultra-fine nano-crystals, which in turn can stabilize emulsion in a better fashion. As for the role of PVA in MOFs co-stabilized C/W HIPEs, it can be considered that, on the one hand, the modification of Ca-BDC particles by PVA molecules through hydrogen bonding and coordination can improve the hydrophilicity (**Figure S1B (bottom)**) of Ca-BDC particles and PVA as glue can network the Ca-BDC particles, on the other hand, the PVA molecules can increase the viscosity of HIPEs[[4](#_ENREF_4), [5](#_ENREF_5)]. Ultimately, under the joint action of PVA molecules and MOF particles, robust protective barriers are formed among HIPEs droplets to effectively preventing the emulsion droplets from coalescence and demulsifying, therefore the HIPEs droplets are more uniform and stable.

**Table S1.** The formulas of C/W HIPEs**^a^**.

| Entries ***^b^*** | PVA(g) | H_2_O(mL) | AM(g) | HEMA(g) | MBMA(g) | Ca-BDC(g) | CO_2_(g) | Bulk density (g/cm^-3^) |
| --- | --- | --- | --- | --- | --- | --- | --- | --- |
| P_0.15_C_50_W_15_ | 0.75 | 15 | 2.506 | 2.294 | 1.2 | 0.15 | 50 | 0.223 |
| P_0.45_C_50_W_15_ | 0.75 | 15 | 2.506 | 2.294 | 1.2 | 0.45 | 50 | 0.202 |
| P_0.75_C_50_W_15_ | 0.75 | 15 | 2.506 | 2.294 | 1.2 | 0.75 | 50 | 0.151 |
| P_0_C_50_W_15_ | 0.75 | 15 | 2.506 | 2.294 | 1.2 | 0 | 50 | 0.256 |
| P_0.45_C_50_W_12_ | 0.45 | 12 | 2.506 | 2.294 | 1.2 | 0.45 | 50 | 0.455 |
| P_0.45_C_50_W_20_ | 0.45 | 20 | 2.506 | 2.294 | 1.2 | 0.45 | 50 | 0.152 |
| P_0.45_C_50_W_15_ | 0.45 | 15 | 2.506 | 2.294 | 1.2 | 0.45 | 50 | 0.158 |
| Control | 0.45 | 15 | 2.506 | 2.294 | 1.0 | 0.45 | 50 | 0.202 |
| Control | 0.45 | 15 | 2.506 | 2.294 | 0.8 | 0.45 | 50 | 0.285 |
| Control | 0.45 | 15 | 2.506 | 2.294 | 0.6 | 0.45 | 50 | 0.352 |
| Control | 0.45 | 15 | 4.8 | 0 | 1.2 | 0.45 | 50 | 0.240 |
| P_0.45_C_40_W_20_ | 0.45 | 20 | 2.506 | 2.294 | 1.2 | 0.45 | 40 | 0.124 |
| P_0.45_C_55_W_20_ | 0.45 | 20 | 2.506 | 2.294 | 1.2 | 0.45 | 55 | 0.163 |
| P_0.45_C_70_W_20_ | 0.45 | 20 | 2.506 | 2.294 | 1.2 | 0.45 | 70 | 0.206 |
| Control | 0.45 | 15 | 1.7 | 3.1 | 1.2 | 0.45 | 50 | 0.357 |
| P_3_C_50_W_15_ | 0.45 | 15 | 2.506 | 2.294 | 1.2 | 3 | 50 | 0.158 |

**^a^Reaction conditions.** All the C/W HIPEs were polymerized by initiation with K_2_S_2_O_8_ (2 wt% relative to monomers) at 60 °C, 150-250 bar, for 10 h.**^b^**PxCyWzwas the abbreviation of polyHIPEs， x, y and z represent the content of Ca-BDC, CO_2_ and water in corresponding C/W HIPEs, repectively.







**B**

**A**

**Figure S****3.** (A), (B) compression stress-strain curves of P**_0.45_**C**_50_**W**_X_** and P**_0.45_**C**_x_**W**_20_** templated from C/W HIPEs containing different water and CO_2_, respectively;

**Table S2.** The micrographs of HepG2 cell proliferation in all groups (200×).

|  | | 48 h | 96 h |
| --- | --- | --- | --- |
| Blanks | | 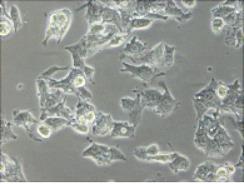 | 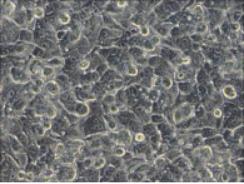 |
| P_0_C_50_W_15_ | 5 mg/ml | 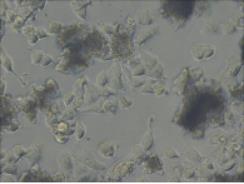 | 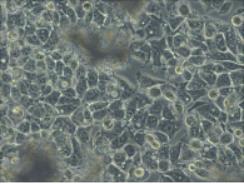 |
|  | 10mg/ml | 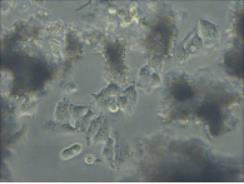 | 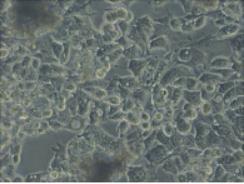 |
|  | 20 mg/ml | 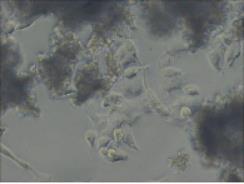 | 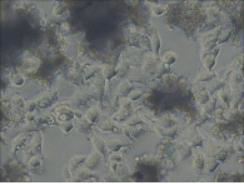 |
| P_0.45_C_50_W_15_ | 5 mg/ml | 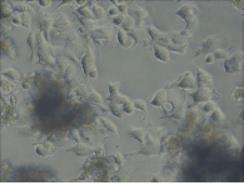 | 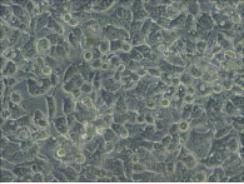 |
|  | 10 mg/ml | 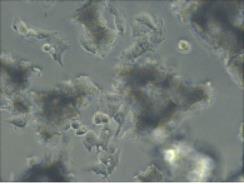 | 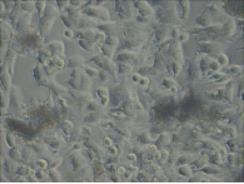 |
|  | 20mg/ml | 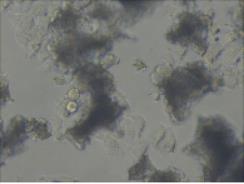 | 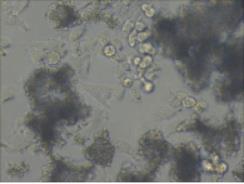 |

**Table S3.** The OD***_450 nm_*** values of HepG2 cell proliferation in all groups.

| Entries | OD***_450 nm_***values | |
| --- | --- | --- |
|  | 48h | 96h |
| Blanks | 0.253±0.002 | 1.005±0.004 |
| P_0_C_50_W_15_ 5mg/ml | 0.253±0.002 | 1.002±0.002 |
| P_0_C_50_W_15_ 10mg/ml | 0.253±0.001 | 0.954±0.005 |
| P_0_C_50_W_15_ 20mg/ml | 0.253±0.002 | 0.852±0.006 |
| P_0.45_C_50_W_15_ 5mg/ml | 0.253±0.002 | 1.005±0.004 |
| P_0.45_C_50_W_15_ 10mg/ml | 0.253±0.001 | 0.899±0.005 |
| P_0.45_C_50_W_15_ 20mg/ml | 0.253±0.003 | 0.649±0.005 |

**Table S4.** The OD***_600nm_*** values of *E. coli* proliferation in all groups.

| Time (h) | OD***_600 nm_***values | | |
| --- | --- | --- | --- |
|  | P0.75C50W15 | Ca-BDC | Blanks |
| 0 | 0.044±0.001 | 0.095±0.001 | 0.095±0.001 |
| 5 | 0.283±0.004 | 0.366±0.004 | 0.358±0.005 |
| 10 | 0.429±0.001 | 0.408±0.002 | 0.43±0.001 |
| 15 | 0.452±0.006 | 0.425±0.006 | 0.49±0.004 |
| 25 | 0.397±0.002 | 0.399±0.002 | 0.462±0.002 |
| 30 | 0.334±0.002 | 0.343±0.002 | 0.389±0.007 |
| 35 | 0.244±0.002 | 0.3±0.006 | 0.306±0.006 |
| 40 | 0.143±0.003 | 0.271±0.005 | 0.283±0.005 |

**Optimal immobilization** **condition and catalytic conditions determination of β-amylase immobilization**


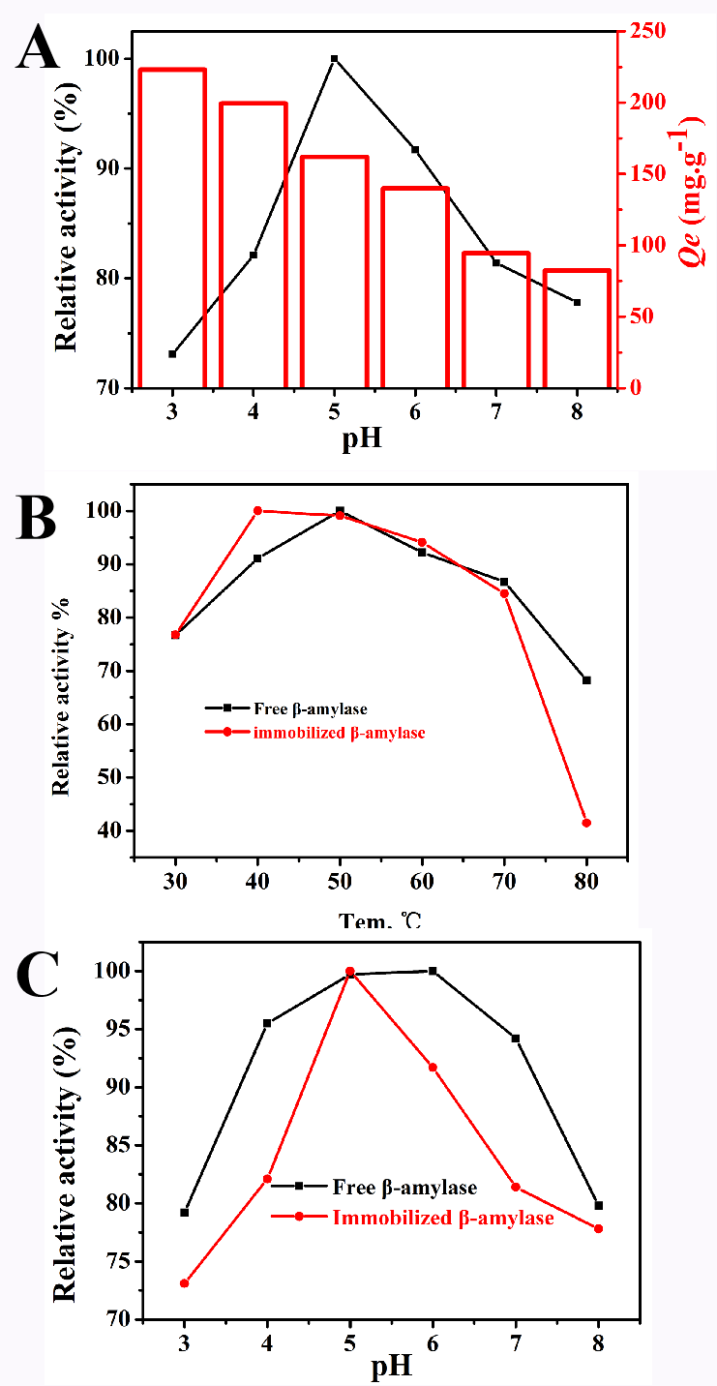


**Figure S****4.** (A) Immobilizing β-amylases into P_0.75_C_50_W_15_ conducted under enzyme solutions with different pH; (B) β-amylases and immobilized ones (prepared by P_0.75_C_50_W_15_ in enzyme solution (1.4 mg/ml) with pH=5) work in starch solutions at different temperature (pH=5); (C) Free β-amylases and immobilized ones (prepared by P_0.75_C_50_W_15_ in enzyme solution (1.4 mg/ml) with pH=5) work in starch solutions with different pH (50°C);

**Figure S4A** shows that, immobilizing β-amylases in enzyme solutions with various pH, there was an optimal pH=5, in which the immobilized ones have the highest enzyme catalytic activity. The free β-amylases and immobilized ones, which were prepared by P**_0.15_**C**_50_**W**_15_** at optimal conditions, worked in starch solutions during 30-80 °C to compare and determine their temperature change resistance. As shown in **Figure S4B**, when temperature is lower than 70°C, the immobilized β-amylase has higher relative catalytic activity compared to free β-amylase. The reason is that (**ⅰ)** the presence of Ca-BDC nano-rods, (**ⅱ)** interconnected macroporous structure and (**ⅲ)** large specific surface area of Ca-BDC/P(AM-*co*-HEMA)HIPEs are beneficial to the substrate diffusion and protection of optimal enzyme conformation, as well the interaction of immobilized β-amylases with substrate under lower relative temperature. The catalytic performances under different pH environments of free β-amylases and immobilized ones were also carried out in starch solutions with different pH to show in **Figure S4C**. There was an optimal pH=5, where free β-amylases and immobilized ones had the highest enzyme activity. The pH range available of immobilized β-amylases is narrower than that of free β-amylases. It can be interpreted that, for immobilized one, the loss of enzyme activity, under acidic conditions, resulted from the part decomposition of Ca-BDC, under alkaline conditions, was ascribed to desorption of part β-amylases into solution and losing its activity due to the negative charge of both Ca-BDC/P(AM-*co*-HEMA)HIPE and β-amylase.

1. M. Mazaj and N. Z. Logar, Phase Formation Study of Ca-Terephthalate MOF-Type Materials, Crystal Growth & Design 15 (2015) 617-624.

2. P. C. Liang, H. K. Liu, C. T. Yeh, C. H. Lin and V. Zima, Supramolecular Assembly of Calcium Metal-Organic Frameworks with Structural Transformations, Crystal Growth & Design 11 (2011) 699-708.

3. C. Liu, J. Zhang, L. Zheng, J. Zhang, X. Sang, X. Kang, B. Zhang, T. Luo, X. Tan and B. Han, Metal–Organic Framework for Emulsifying Carbon Dioxide and Water, Angewandte Chemie 55 (2016) 11372-11376.

4. S. Zou, Z. Wei, H. Yang, Y. Deng and C. Wang, Macroporous antibacterial hydrogels with tunable pore structures fabricated by using Pickering high internal phase emulsions as templates, Polymer Chemistry 5 (2014) 4227-4234.

5. H. Zhu, Q. Zhang and S. Zhu, Assembly of Metal-Organic Framework into 3D Hierarchical Porous Monoliths through Pickering High Internal Phase Emulsion Template, Chemistry 22 (2016) 8751-8755.
